# Male Bowhead Whale Reproductive Histories Inferred from Baleen Testosterone and Stable Isotopes

**DOI:** 10.1093/iob/obac014

**Published:** 2022-05-04

**Authors:** Kathleen E Hunt, C Loren Buck, Steven H Ferguson, Alejandro Fernández Ajo, Mads Peter Heide-Jørgensen, Cory J D Matthews

**Affiliations:** Smithsonian-Mason School of Conservation & Department of Biology, George Mason University, 1500 Remount Rd, Front Royal, VA 22630, USA; Department of Biological Sciences, Northern Arizona University, 617 S. Beaver St., Flagstaff, AZ 86011, USA; Fisheries and Oceans Canada, Arctic Aquatic Research Division, 501 University Crescent, Winnipeg, MB R3T 2N6, Canada; Marine Mammal Institute, Fisheries and Wildlife Department, Oregon State University, Newport, OR 97365, USA; Greenland Institute of Natural Resources, Strandgade 91,2, DK-1401 Copenhagen K, Denmark; Fisheries and Oceans Canada, Arctic Aquatic Research Division, 501 University Crescent, Winnipeg, MB R3T 2N6, Canada

## Abstract

Male mammals of seasonally reproducing species typically have annual testosterone (T) cycles, with T usually peaking during the breeding season, but occurrence of such cycles in male mysticete whales has been difficult to confirm. Baleen, a keratinized filter-feeding apparatus of mysticetes, incorporates hormones as it grows, such that a single baleen plate can record years of endocrine history with sufficient temporal resolution to discern seasonal patterns. We analyzed patterns of T every 2 cm across the full length of baleen plates from nine male bowhead whales (*Balaena mysticetus*) to investigate occurrence and regularity of T cycles and potential inferences about timing of breeding season, sexual maturation, and reproductive senescence. Baleen specimens ranged from 181–330 cm in length, representing an estimated 11 years (smallest whale) to 22 years (largest whale) of continuous baleen growth, as indicated by annual cycles in stable isotopes. All baleen specimens contained regularly spaced areas of high T content (T peaks) confirmed by time series analysis to be cyclic, with periods matching annual stable isotope cycles of the same individuals. In 8 of the 9 whales, T peaks preceded putative summer isotope peaks by a mean of 2.8 months, suggesting a mating season in late winter / early spring. The only exception to this pattern was the smallest and youngest male, which had T peaks synchronous with isotope peaks. This smallest, youngest whale also did not have T peaks in the first half of the plate, suggesting initiation of T cycling during the period of baleen growth. Linear mixed effect models suggest that whale age influences T concentrations, with the two largest and oldest males exhibiting a dramatic decline in T peak concentration across the period of baleen growth. Overall, these patterns are consistent with onset of sexual maturity in younger males and possible reproductive senescence in older males. We conclude that adult male bowheads undergo annual T cycles, and that analyses of T in baleen may enable investigation of reproductive seasonality, timing of the breeding season, and life history of male whales.

## Inuktitut

ᐊᑎᖓ:

ᐊᖑᑏ ᐊᕐᕕᑦ ᐆᒪᔪᓕᐅᒃᑲᓂᓂᕐᒧᑦ ᐱᖅᑯᓯᑐᖃᖏᑦ ᓱᖅᑲᓂᑦ testosterone ᐊᒻᒪ stable isotopes

ᓇᓗᓇᐃᖅᓯᒪᓪᓚᕆᖏᑦᑐᑦ

ᐊᖑᑎᑦ ᐆᒪᔪᐃᑦ ᐊᕐᕌᒍ ᐃᓗᐊᓂ ᐆᒪᔪᓕᐅᒃᑲᓐᓂᖃᑦᑕᖅᑐᑦ ᐆᒪᔪᐃᑦ ᐊᕐᕌᒍᑕᒫᑦ ᐱᑕᖃᖅᓕᖅᐸᑦᑐᑦ testosterone ᐅᑎᖅᑕᖃᑦᑕᕐᓂᖏᑦ, testosterone ᖁᕝᕙᓯᓕᖃᑦᑕᖅᑐᑎ ᓄᓕᐊᓕᖅᑎᓪᓗᒋᑦ, ᑭᓯᐊᓂ ᓴᖅᑭᖃᑦᑕᕐᓂᖏᑦ ᐅᑎᖅᑕᖃᑦᑕᖅᑐᓂ ᐊᖑᑎᓂᑦ ᐊᕐᕕᓂᑦ ᐱᒡᒐᓇᖅᓯᒪᔪᖅ ᓇᓗᓇᐃᕐᓗᒍ.. Baleen, ᓂᕆᔾᔪᑎᖏᑦ ᓱᖅᑲᓕᓐᓂ ᕿᓚᓗᒐᕐᓂᑦ, ᐱᖃᓯᐅᔾᔨᔪᑦ hormones ᐱᕈᖅᐸᓪᓕᐊᑎᓪᓗᒍ, ᐊᑕᐅᓯᑐᐃᓐᓇᖅ ᓱᖅᑲᖅ ᓇᓗᓇᐃᖅᓯᔪᓐᓇᖅᑐᑦ ᐊᕐᕌᒍᒐᓚᓐᓂᑦ hormone ᐱᖅᑯᓯᑐᖃᖏᓐᓂ ᓈᒻᒪᓐᓂᖃᖅᑐᑎ ᖃᐅᔨᓴᕈᑎᒋᓪᓗᒍ ᐊᕐᕌᒍ ᐃᓗᐊᓂ ᖃᓄᐃᓕᖓᖃᑦᑕᕐᓂᖏᓐᓄᑦ. ᖃᐅᔨᓴᓚᐅᖅᑕᕗᑦ ᖃᓄᐃᓕᖓᖃᑦᑕᕐᓂᖏᑦ testosterone ᐅᑯᓇᓂᑕᒫᖅ 2 cm ᑕᑭᓂᖓᓄ ᐊᖑᑎᓂᑦ ᖁᓕᐅᖏᒑᖅᑐᓂᑦ ᐊᕐᕕᓂᑦ (*Balaena mysticetus*) ᖃᐅᔨᓴᕐᓗᒍ ᓴᖅᑭᖃᑦᑕᕐᓂᖏᑦ ᐊᒻᒪ ᓴᖅᑭᖃᑦᑕᕋᔪᓐᓂᖏᑦ testosterone ᐅᑎᖅᑕᕐᓂᖏᑦ ᐊᒻᒪ ᖃᓄᐃᓕᖃᖅᑎᑦᑎᔪᓐᓇᕐᓂᖏᑦ ᖃᖓᒃᑰᓂᖓᓄ ᓄᓕᐊᖃᑦᑕᑎᓪᓗᒋᑦ, ᐆᒪᔪᓕᐅᕈᓐᓇᕐᓗᑎ ᐱᕈᕐᓂᖏᑦ, ᐊᒻᒪ ᐆᒪᔪᓕᐅᒃᑲᓂᓂᕐᒧᑦ ᐊᕐᕌᒍᖃᓕᖅᐸᓪᓕᐊᓂᖏᑦ. ᓱᖅᑲᖏᓐᓂ ᐊᑯᓐᓂᖏᓐᓂ ᐱᒋᐊᖅᑐᑎ 181-330 cm ᑕᑭᓂᖏᑦ, ᑭᒡᒐᖅᑐᐃᔪᑦ ᒥᔅᓴᐅᓴᑦᑕᐅᓯᒪᔪᑦ 11 ᐊᕐᕌᒍᐃᑦ (ᒥᑭᓛᖅ ᐊᕐᕕᒃ) ᑎᑭᑦᑐᒍ 22 ᐊᕐᕌᒍᓄᑦ (ᐊᖏᓛᑦ ᐊᕐᕖᑦ) ᓱᖅᑲᖏᑦ ᐱᕈᖅᐸᓪᓕᐊᖏᓐᓇᖅᑐᑦ, ᓇᓗᓇᐃᖅᑕᐅᓯᒪᔪᑦ ᐊᕐᕌᒍᑕᒫᑦ ᐅᑎᖅᑕᖃᑦᑕᕐᓂᖏᓐᓄᑦ stable isotopes. ᐊᑕᖏᖅᑐᒋᑦ ᓱᖅᑲᐃᑦ ᐃᓗᐊᓃᑎᑕᐅᔪᑦ ᐊᑯᓕᕇᖅᑐᑎᑦ ᖃᖓᒃᑰᖃᑦᑕᕐᓂᖏᓐᓄᑦ testosterone ᐅᑎᖅᑕᖃᑦᑕᕐᓂᖏᑦ ᖃᖓᒃᑰᓂᖏᑦ ᒪᓕᓐᓂᖃᖅᑐᑎ ᐊᕐᕌᒍᑕᒫᑦ ᐱᕈᕐᓂᖏᓐᓄᑦ. ᑎᓴᒪᐅᔪᑦ ᖁᓕᐅᖏᒑᖅᑐᓂᑦ ᐊᕐᕕᑦ, testosterone ᖁᕝᕙᕆᐊᖃᑦᑕᖅᑐᑦ ᐊᐅᔭᕈᓐᓂᐊᓕᖅᑎᓪᓗᒍ isotope ᖁᕝᕙᕆᐊᖅᑐᑎ ᐊᑐᖅᑕᐅᒐᔪᑦᑐᑦ 2.8 ᑕᖅᑮᑦ, ᓇᓗᓇᐃᖅᓯᔪᖅ ᓄᓕᐊᕐᓂᐅᔪᑦ ᐅᑭᐅᒃᑯᑦ ᓄᖑᖏᓐᓂ / ᐅᐱᕐᖔᕋᑖᒥ. ᑭᓯᐊᓂᓕ ᑕᕝᕙᓂ ᐊᑐᖅᑕᐅᕙᑦᑐᓂᑦ ᒥᑭᓛᖑᓚᐅᖅᑐᖅ ᐊᒻᒪ ᒪᒃᑯᓛᖑᓪᓗᓂ ᐊᖑᑎ, ᐱᑕᖃᓚᐅᖏᑦᑐᑦ testosterone ᖁᕝᕙᕆᐊᕐᓂᖏᓐᓂ ᓯᕗᓪᓕᖅᐹᖓᓂ ᓇᑉᐸᖓᓂ ᓱᖅᑲᖓ, ᓇᓗᓇᐃᖅᓯᓂᖃᖅᑐᑦ ᐱᒋᐊᖅᑎᑕᐅᓂᖓ testosterone ᐅᑎᖅᑕᖃᑦᑕᕐᓂᖓ ᓱᖅᑲᖏᑦ ᐱᕈᖅᐸᓪᓕᐊᑎᓪᓗᒋᑦ. ᑭᓪᓕᓯᓂᐊᖅᑕᐅᓯᒪᔪᑦ ᖃᐅᔨᓴᖅᑕᐅᓂᖏᑦ ᓇᓗᓇᐃᖅᓯᔪᑦ ᐊᕐᕖᑦ ᐊᕐᕌᒍᖏᑦ ᐊᔭᐅᖅᓯᒋᐊᕐᓂᖃᖅᑐᑦ testosterone ᐱᑕᖃᕐᓂᖏᓐᓄᑦ, ᒪᕐᕉᒃ ᐊᖏᓛᒃ ᐊᒻᒪ ᐃᓐᓇᐅᓛᒃ ᐊᖑᑏᒃ ᑕᑯᔅᓴᐅᑎᑦᑎᔪᑦ ᐊᖏᔪᒥᑦ ᐊᑦᑎᓪᓕᒋᐊᖅᑐᓂᑦ testosterone ᐱᑕᖃᕐᓂᖏᓐᑦ ᐊᑯᓂᐅᓂᖓᓄ ᓱᖅᑲᖏ ᐱᕈᕐᓂᖏᓐᓄᑦ. ᐊᑕᖏᓕᒫᖅᑐᒍ, ᑖᒃᑯᐊ ᖃᓄᐃᓘᖃᑦᑕᕐᓂᖏᑦ ᒪᓕᓐᓂᖃᖅᑐᑦ ᓇᕝᕕᖏᓐᓂ ᓄᓕᐊᕈᓐᓇᓯᓪᓗᑎ ᐊᕐᕌᒍᑭᓐᓂᖅᓴᓂᑦ ᐊᖑᑎᓂᑦ ᐊᒻᒪ ᐆᒪᔪᓕᐅᒃᑲᓐᓂᕈᓐᓇᖅᓯᓗᑎ ᐊᕐᕌᒍᖏᑎᒍᑦ ᐊᕐᕌᒍᖃᕐᓂᖅᓴᐅᔪᓂᑦ ᐊᖑᑎᓂᑦ. ᓄᖅᑲᐅᓯᖅᑐᒍᑦ ᑖᒃᑯᐊ ᐊᖑᑏᑦ ᐊᕐᕖᑦ ᐊᕐᕌᒍᑕᒫᑦ testosterone ᐅᑎᖅᑕᖃᑦᑕᖅᑐᑦ, ᐊᒻᒪ ᑖᒃᑯᐊ ᖃᐅᔨᓴᐃᓂᐅᔪᑦ testosterone ᓱᖅᑲᖏᓐᓂ ᖃᐅᔨᓴᕈᓐᓇᑎᑦᑎᓂᐊᖅᑐᑦ ᐆᒪᔪᓕᐅᒃᑲᓂᕐᓂᖏᓐᓄᑦ ᐊᕐᕌᒍ ᐃᓗᐊᓂ, ᖃᖓᒃᑰᓂᖓ ᐆᒪᔪᓕᐅᕐᓂᐅᔪᑦ ᐊᕐᕌᒍ ᐃᓗᐊᓂ, ᐊᒻᒪ ᐆᒪᓂᖏᓐᓄᑦ ᐱᑯᓯᑐᖃᖏᑦ ᐊᖑᑏᑦ ᐊᕐᕖᑦ.

## Danish (Dansk)

Hanner af pattedyr med faste sæsoner for reproduktion har typisk årlige testosteron (T) cyklusser, hvor T normalt topper i ynglesæsonen. Forekomsten af ​​sådanne cyklusser har dog været vanskelig at bekræfte hos bardehvaler. Barder som er et keratiniseret filtreringsapparat hos bardehvaler, indlagrer hormoner efterhånden som barderne vokser, således at en enkelt barde kan indeholde mange års endokrin historie med en tidsmæssig opløsning som er tilstrækkelig til at skelne sæsonbestemte mønstre. Vi analyserede mønstre og cyklusser af T for hver 2 cm på tværs af den fulde længde af barder fra ni grønlandshvaler (Balaena mysticetus). Formålet var at observere tidspunktet for ynglesæsonen, alder ved kønsmodenhed og eventuel reproduktiv senilitet. Barde prøverne varierede fra 181 til 330 cm i længden, hvilket repræsenterer omtrentlige aldre mellem 11 år (mindste hval) og 22 år (største hval) med kontinuerlig bardevækst, som observeret i de årlige cyklusser i stabile isotoper. Alle barde-prøverne indeholdt regelmæssigt adskilte områder med højt T-indhold (T-toppe), bekræftet af tidsserieanalyser som værende cykliske, med perioder, der matchede årlige stabile isotopcyklusser for de samme individer. Hos otte af de ni hvaler gik T-toppene forud for formodede sommerisotop-toppe med et gennemsnit på 2,8 måneder, hvilket tyder på en parringssæson sidst på vinteren/det tidlige forår. Den eneste undtagelse fra dette mønster var den mindste og yngste han, som havde T-toppe synkront med isotoptoppene. Denne mindste, yngste hval havde heller ikke T-toppe i den første halvdel af barden, hvilket tyder på initiering af T-cyklus i perioden med barde-vækst. Lineære effekt modeller tyder på, at hvalens alder påvirker T-koncentrationerne, hvor de to største og ældste hanner udviser et dramatisk fald i T-top koncentrationen i hele perioden med bardevækst. Generelt er disse mønstre i overensstemmelse med begyndende seksuel modenhed hos yngre hanner og mulig reproduktiv senilitet hos ældre hanner. Vi konkluderer, at voksne grønlandshvaler gennemgår årlige T-cyklusser, og at analyser af T i barder kan bruges til at undersøge sæsonen for reproduktionen, tidspunktet for parringerne og han-hvalernes generelle livshistorie.

## Spanish

Los individuos macho en especies de mamíferos que se reproducen estacionalmente suelen presentar ciclos anuales de testosterona (T), la cual usualmente alcanza un pico de concentración durante la temporada de reproducción. La ocurrencia de dichos ciclos anuales no ha sido evidenciada en los misticetos. Las barbas, estructuras queratinizadas que conforman el aparato de filtración de los misticetos, incorporan hormonas a medida que crecen. De esto resulta que en cada barba de un individuo existe un registro continuo de su historia endócrina que puede abarcar años, y presentar suficiente resolución temporal para discernir patrones estacionales. En este trabajo analizamos los patrones de T cada 2 cm a lo largo de las barbas de nueve individuos machos de ballenas de Groenlandia (*Balaena mysticetus*) e investigamos la ocurrencia y regularidad de los ciclos de T para inferir sobre la temporalidad de reproducción, la madurez sexual y la senescencia reproductiva. Las muestras de barbas utilizadas en este estudio oscilaron entre 181 y 330 cm de longitud, representando un registro continuo que abarca aproximadamente 11 años (para la ballena más pequeña) y 22 años (para la ballena más grande), según los ciclos anuales en isótopos estables observados. Todos los especímenes de barbas mostraron áreas regularmente espaciadas con alto contenido de T (picos de T), confirmados como cíclicos mediante un análisis de series de tiempo, y presentan períodos que coinciden con los ciclos anuales de isótopos estables para los mismos individuos. En ocho de las nueve ballenas, los picos de T precedieron a los supuestos picos de isótopos de verano en una media de 2,8 meses. Esto sugiere que la temporada de apareamiento ocurre hacia fines del invierno o principios de la primavera. Una excepción para este patrón fue lo estimado para el macho más pequeño y más joven, en el cual los picos de T fueron sincrónicos con los picos de isótopos. Este individuo, el más pequeño y joven, tampoco presentó picos de T en la primera mitad de la barba, lo que sugiere que el inicio de los ciclos de T ocurrió durante el período que abarca el crecimiento de la barba. Los modelos lineales mixtos indican que la edad de las ballenas influye en las concentraciones de T, observándose una marcada disminución en la concentración máxima de T durante el período de crecimiento de las barbas en los dos individuos machos de mayor tamaño y edad. En general, estos patrones son consistentes con el inicio de la madurez sexual en los machos más jóvenes y posiblemente con la senescencia reproductiva en los machos de mayor edad. Concluimos que los machos adultos de ballenas de Groenlandia presentan ciclos anuales de T, y que los análisis de T en las barbas permiten la investigación de la estacionalidad reproductiva, el momento de la temporada de reproducción y la historia de vida de las ballenas macho.

## Introduction

The vertebrate steroid hormone testosterone (T) mediates several key physiological and behavioral processes of male reproduction (Hau 2007; Lipshutz et al. 2019). T is necessary for spermatogenesis, and often facilitates reproductive behaviors such as courtship, mating activity, and male-male competition (Dixson and Anderson 2004; Hau 2007). In seasonally breeding mammals, T typically shows an annual pattern with a sharp seasonal peak coincident with spermatogenesis and mating behavior (Hau 2007). In addition to such seasonal patterns, there are also often pronounced individual differences in T concentrations, sometimes related to age or life stage. For example, in many species, immature males exhibit low or undetectable circulating T that rises sharply at the onset of sexual maturity, and then, in some species, declines in the oldest males (reproductive senescence; Beehner et al. 2009; Chen et al. 2009). These age effects combine with multiple other influences on T (e.g., social cues, stress, body condition, experience, dominance, *etc.*; Reeder and Kramer 2005; Greiner et al. 2010; Muehlenbein and Watts 2010; Ball and Balthazart 2020; Moore et al. 2020; Dalle Luche et al. 2021; Tomiyasu et al. 2021), resulting in considerable individual variation in hormone patterns across time.

T patterns have been well-documented in short-lived terrestrial species such as passerine birds and small mammals, as well as some intermediate-sized terrestrial mammals (Buck and Barnes 2003; Muller 2017; Wingfield et al. 2020; Gomes et al. 2021). In large and long-lived species, however, particularly marine species, measuring T across seasons and life stages is complicated by the difficulty of serial sampling of individuals. The mysticete (baleen-bearing) whales are perhaps the least understood; lack of a viable method for live-capture or repeated blood sampling has challenged ability to investigate potential seasonal reproduction as well as life history influences on reproductive function (Hunt et al. 2013). Most mysticetes seasonally migrate and have a well-defined calving season, strongly suggestive of seasonal breeding, but mating locations remain unidentified for some species and little data exist to confirm whether adults, particularly males, might have annual cycles of reproductive hormones. In recent years, the development of methods for hormone extraction and quantification from several non-plasma sample types has offered a new avenue of investigation into baleen whale physiology (Hunt et al. 2013). Hormone concentrations in blubber biopsies and fecal samples, as well as changes in testicular histology, suggest seasonal changes in T in males of several species (Cates et al. 2019; Tarpley et al. 2021), but repeated samples have not been obtained from the same males over time, and sampling is usually limited to certain months of the year. Endocrine analysis of layers of cerumen (earwax) have revealed important clues as to the age of onset of sexual maturity in a case study of a male blue whale (*Balaenoptera musculus)*; however, the temporal resolution of earplug endocrine data limits ability to detect seasonal patterns (Trumble et al. 2013). Longitudinal data from male whales with sufficient temporal resolution to separate seasons would be valuable not only for study of male reproductive biology in mammals generally, but also for conservation and management purposes (i.e., identification of critical habitat and timing of reproduction).

Analysis of endocrine patterns in baleen offers a potential avenue for such research. Baleen, the keratinous filter-feeding apparatus of mysticete whales, grows continuously from the upper palate while simultaneously wearing distally (Werth and Sformo 2021). Steroid and thyroid hormones are deposited in baleen as it grows, such that a complete baleen plate contains a continuous retrospective record of the whale's endocrine history spanning the time period of baleen growth, over a decade in bowhead whales (*Balaena mysticetus*) (Hunt et al. 2014, 2016, 2017a, 2018; Rolland et al. 2017; Lysiak et al. 2018). Temporal context of endocrine profiles can be determined via analysis of ratios of stable isotopes (SI) in baleen (e.g., δ^15^N), which in many species (including bowhead whales) show annual oscillations reflecting migrations between summer and winter ranges (Schell et al. 1989a, 1989b; Matthews and Ferguson 2015). Though the rate of deposition of circulating hormones into growing baleen remains to be determined, comparison of baleen endocrine and stable isotope patterns to known dates of individually documented events (e.g., date of parturition, date of exposure to a stressor, movement of tagged individuals between seasonal habitats, etc.) suggest an approximately ∼2–4 week resolution for both baleen endocrine data and stable isotope data (Schell et al. 1989b, 1989a; Hunt et al. 2016, 2017a; Fernández Ajó et al. 2018, 2020; Lowe et al. 2021). Thus, baleen captures a multi-year timeframe, but also has sufficient temporal resolution to determine seasonal endocrine patterns.

Baleen hormone profiles have been utilized to explore pregnancy-related patterns in female whales (Hunt et al. 2016; Rolland et al. 2017; Lysiak et al. 2018), but data on males have been restricted to a single pilot study including only one individual from each of three whale species (blue whale, bowhead whale, and North Atlantic right whale [*Eubalaena glacialis*]; Hunt et al. 2018). Although limited in scope, the pilot analysis revealed regularly spaced areas of high T content suggestive of annual T cycles in baleen of each of the three individuals. Here, we focus on bowhead whales for further study, due to the fact that bowheads have exceptionally long (up to 3–4 m) and slow-growing baleen that captures time frames spanning 10–25 years in adults (Lubetkin et al. 2008; Werth and Sformo 2021). Annual baleen growth rates range from 16–25 cm/yr in bowhead whales (with a predictable slowing with age), and plates can readily be sampled at sub-seasonal resolution (Schell et al. 1989a). Bowheads are also the only Arctic-endemic baleen whale, having evolved unique adaptations to the seasonal extremes of their environment (e.g., variation in sea ice extent, ambient light levels, food availability), including long lifespans that can exceed 200 yr (George and Bockstoce 2008; George et al. 2021). These features make the bowhead whale an interesting model species in which to further investigate T patterns with respect to reproductive seasonality and life history (e.g., possible influences of age on reproduction), which have been difficult to assess in this and other large whale species.

In this study, we add eight additional male bowhead whales to the single male presented in the pilot study of Hunt et al. (2018), assessing T patterns along the entire length of baleen of all nine whales. We combine T data with previously published stable isotope (SI) data from the same individuals to address the following questions: (1) Do longitudinal T profiles in baleen of male bowhead whales exhibit annual cycles consistent with seasonal reproduction (i.e., are T peaks narrow—occurring only in part of the year—and regularly spaced, with periods matching those of SI cycles)? (2) If so, does the timing of T cycles allow potential inferences about the timing of the breeding season (i.e., are annual elevations in T, if they occur at all, brief enough to be temporally linked to a certain season)? (3) Do patterns in T differ with age? We provide evidence that the answer to all three questions is yes, and discuss baleen T profiling as a new approach that may enable study of seasonality and location of mysticete whale mating, as well as other aspects of male reproductive life history, with potential implications for conservation of these large marine vertebrates.

## Methods

### Baleen collection

**Fig. 1:**
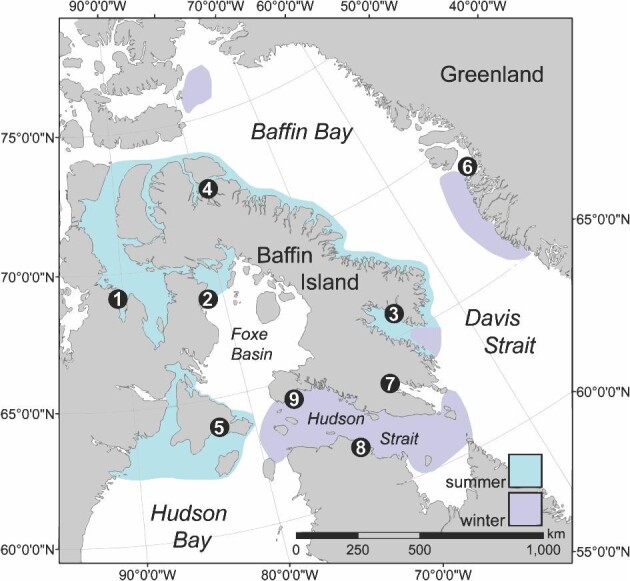
Collection locations of baleen samples from nine male Eastern Canada-West Greenland (EC-WG) bowhead whales (*Balaena mysticetus*) in the Eastern Canadian Arctic and West Greenland. Individual whale locations are shown with black circles, with whales numbered from 1 to 9 in order of best available estimate of age, *i.e.* matching whale numbers in Table 1. Core summer and winter use areas of the EC-WG bowhead whale population is indicated by aqua (summer) and lavender (winter) shading.

Baleen was collected from nine male bowhead whales harvested in Inuit indigenous hunts in the eastern Canadian Arctic (*n* = 8) and Greenland (*n* = 1) from 1998–2011 (Table 1; Fig. 1). Entire plates were collected for seven of the whales, while baleen from two whales was cut at the gumline and therefore lacked approximately 20–26 cm of most recently grown baleen embedded within gum tissue (approximately one year of growth; Schell et al. 1989a; Lubetkin et al. 2008). Baleen was collected within 24–48 h of death, cleaned of soft tissue at the time of necropsy, and stored at –25°C until sampling. Before sampling, algae and any other adhered material were removed with water and scrubbing pads, and the baleen was then scraped with a scalpel to remove surface baleen, exposing untouched baleen for sampling (see Matthews & Ferguson 2015).

**Table 1:** Collection and specimen data for the nine bowhead whales of this study. Whales are numbered in order of estimated age, younger to older, using best available age estimation method (aspartic acid racemization [AAR] if available, body length if not). All whales were confirmed male at necropsy.

Specimen	Whale field identification code	Collection location	Collection date (mo/yr)	Body length (m)	Baleen length (cm)^c^	Age estimated from body length (yr)^d^	Age estimated from AAR of eye lens (yr)^e^
1	BM-NSA-2008-001	Gulf of Boothia, Kugaaruk, NU, Canada	09/2008	10.51	181	10	14
2	BM-NSA-2008-002	Foxe Basin, Hall Beach, NU, Canada	08/2008	13.43	235	29	18
3	BM-NSA-98-01^a^	Cumberland Sound, Pangnirtung, NU, Canada	07/1998	12.75	266	22	21
4	BM-NSA-2010-01	Eclipse Sound, Pond Inlet, NU, Canada	08/2010	12.80	230	23	*n/a*
5	BM-CH-2000-001	Hudson Bay, Coral Harbor, NU, Canada	08/2000	11.65	270	15	24
6	BH3^b^	Disko Bay, Greenland	04/2009	14.10	204	41	44
7	BM-NSA-2011-01	Frobisher Bay, Iqaluit, NU, Canada	08/2011	14.33	298	48	*n/a*
8	BM-01-2008	Hudson Strait, Kangiqsujuak, QC, Canada	08/2008	14.88	319	78	115
9	BM-NSA-2009-03	Hudson Strait, Cape Dorset, NU, Canada	09/2009	15.77	330	∼153	*n/a*

a Whale “NSA-BM-98-01” of Matthews & Ferguson (2015).

b Whale “322” of Heide-Jørgensen et al. (2012).

c Includes baleen embedded in gum + erupted baleen, except Whales 5 and 6 (for which the plate was cut at the gumline,that is, erupted length only).

d Body length age estimates from von Bertalanffy II two-stage growth model (Whales 1–7), von Bertlanffy 1a single-stage growth model (Whale 8) or *via* average of ages of other known male bowhead whales in same body length class (Whale 9); Lubetkin et al. (2012).

e Age estimates via aspartic acid racemization of the eye lens (Heide-Jørgensen et al. 2012).

### Age estimation

Age in bowhead whales can be estimated through a variety of methods (comprehensively reviewed in George et al. 2021). Currently, the best-validated methods rely on body length or aspartic acid racemization (AAR) rates of the eye lens (Rosa et al. 2011). Body length age estimates are progressively less precise for greater ages, particularly over 60 years when body length approaches an asymptote (George et al. 2021). At present, the recommended body length age estimate equation for bowhead whales is the von Bertalanffy II two-stage growth model with male-bowhead-specific parameters (see Lubetkin et al. 2012, and George et al., 2021), which incorporates two different growth rates, one typical of subadult whales and another for older whales. However, some males exceed the maximum length allowed by the von Bertalanffy II equation; in such cases, an alternate equation (von Bertalanffy Ia single-stage growth model; Lubetkin et al., 2012), or comparison to other whales of similar body length that have been aged by other methods, can provide improved age-estimate precision for these older males. In this study, body lengths were available for all nine whales (Table 1), of which two (Whales 8 and 9) exceeded the body length limit of the von Bertalanffy II two-stage growth model. Whale 8, therefore, was aged using the alternate von Bertalanffy Ia single-stage growth model (Lubetkin et al. 2012), producing an estimated age of 78 years. Whale 9 was too large even for this alternate model—that is, Whale 9’s length was greater than the hypothetical maximum for male bowhead whales—and his age was therefore estimated based on the average of AAR-estimated ages of four other known males with body lengths also exceeding the theoretical maximum (aged at 135, 146, 159, and 172 years; Lubetkin et al. 2012); producing an age estimate of ∼153 years for Whale 9 (Table 1). To investigate any effect of potential inaccuracy in this age estimate of Whale 9, exploratory analyses were also performed using the minimum (135 years) or the maximum (172 years) age estimates for Whale 9, or excluding Whale 9 from analysis entirely; no differences in direction or significance were noted for any result (data not shown).

The AAR rate in the eye lens, which in mammals occurs at a steady rate determined by body temperature, is generally regarded as a superior age-estimation method for bowhead whales (George et al. 2021). AAR is currently the only well-validated method that can provide relatively precise age estimates for older whales, and it can also be used on whales as young as ∼10 years of age (George et al. 2021). AAR-estimated age was not available for all bowhead whales in our sample, however, as it requires collection of an eye lens, which was not always possible given necropsy logistical constraints and field conditions in the Arctic. In this study, AAR-estimated ages were available for six of the nine whales for which the eye lens had been collected at necropsy (Table 1; Heide-Jørgensen et al. 2012, Fisheries & Oceans Canada unpublished data).

Ultimately we conducted two analyses for all questions regarding potential age effects, utilizing either: (1) Best available age estimate, i.e., AAR-derived age estimates for the six whales for which they were available, and body-length-derived age estimates for the three whales for which AAR age estimates were not available (so as to keep sample size at *n* = 9, necessary for acceptable statistical power); and (2) Body length only, i.e., age estimated solely based on body length for all whales (*n* = 9).

### Baleen subsampling

For each whale, the longest baleen plate available (range 181–330 cm; Table 1) was sampled for endocrine analyses as in previously published protocols (Matthews and Ferguson 2015). In brief, starting at the most proximal point (“base”), subsamples of baleen powder were collected using a hand-held drill fitted with a 1/16 inch bit to drill a series of small vertical bore holes (extending partway into the plate, but not fully penetrating through the plate) at sampling locations spaced every 2 cm across the length of the plate. This sampling interval corresponds to an estimated ∼1–2 mo of baleen growth in adult bowhead whales, depending on age-specific growth rate (faster in younger whales, slower in older males; Lubetkin et al. 2008; Matthews and Ferguson 2015). Baleen powder from each sampling location was collected, mixed well, and stored frozen at –25°C in individual vials until further processing.

### Hormone extraction

Hormones were extracted from powdered baleen samples with modifications of prior studies (Hunt et al. 2014, 2016, 2017a, 2017b, 2018; Fernández et al. 2021), as follows: 6.00 mL of 100% HPLC-grade methanol was added to 100 mg of baleen powder in 16×100 mm borosilicate glass tubes, vortexed 2 h, and centrifuged for 15 min at 3000 g. Methanol supernatant (containing hormones) was transferred to a 13×100 mm borosilicate glass tube for dry-down, with results corrected for percentage of supernatant recovered. Supernatants were dried under compressed N_2_ stream for ∼6 h and reconstituted in 500 uL of EIA assay buffer (buffer “X065”, Arbor Assays, Ann Arbor, MI, USA), sonicated 5 min, vortexed 5 min, pipetted to a cryovial, left to sit at 4°C for 30 min (allowing remaining particulates to settle out), and finally decanted to an externally threaded, o-ring-capped cryovial for long-term storage at –80°C. This is termed the “1:1” (full-strength, neat) extract. All extracts were assayed within four months of extraction.

### Hormone assays

Samples were assayed with T enzyme immunoassays that have previously been validated for bowhead baleen (testosterone kit #K032, Arbor Assays, Ann Arbor, MI, USA; Hunt et al. 2017b, 2018). Hormone extracts were diluted to 1:4 to keep results near 50% bound on the assay standard curve, the area of greatest precision (Grotjan and Keel 1996). Each whale's samples were assayed on the same day using the same lots of reagents. Sample order was randomized within and across assay microplates to minimize any influences of intra- or inter-assay variation on longitudinal data. The manufacturer's assay protocol (available at www.arborassays.com) was followed with one modification, extension of the serial dilution of standards to produce one additional low-dose standard, as in Hunt et al. (2018). The final standard curve utilized eight standards spanning 16–10,000 pg/mL. All assays followed standard QA/QC including assay of quadruplicates of all non-specific binding wells and blanks (zero dose); duplicates of all standards, controls and samples; assay of reference controls in every plate; re-assay of any sample with coefficient of variation (CV) >10% between duplicates; exclusion from the standard curve of any single standard with >10% CV; and full re-assay of any assay with more than two anomalous standards (CV >10%), controls outside of normal bounds, or optical density outside of normal bounds. See Hunt et al. (2017b, 2018) for additional assay details including antibody sources, inter- and intra-assay precision, sensitivity, and cross-reactivities. All assay results were converted to nanograms of immunoreactive hormone per g of baleen powder.

### Statistical analysis

Periodicity of each whale's T profile was initially assessed using autocorrelation analysis to identify lags at which T concentrations were autocorrelated. Periods of T cycles were then estimated using spectral analysis of high order autoregressive models (AR[*p*]) fit to data after detrending using a Gaussian low pass filter to remove low frequency variation. AR models were fit using the R function ‘spectrum’ (R Core Team 2021), which determines which order (*p*) model provides the best realization of the time series by minimizing Akaike's Information Criterion (AIC), and then estimates the spectral density for that model. The spectral peak frequency (cycles per sampling interval) of each modeled profile was converted to samples per cycle (1/peak frequency), then multiplied by the 2-cm sample increment to estimate period. Annual cyclicity of T was assessed by cross-correlation analysis of T profiles with previously measured stable nitrogen isotope (δ^15^N) cycles in these same baleen plates (Matthews and Ferguson 2015; Hunt et al. 2018), which are assumed to be annual cycles resulting from the annual migration between (and feeding within) isotopically distinct summer and winter foraging grounds. Putative season of T elevations was assessed via offset (% phase shift) of T peaks from δ^15^N peaks assumed to represent summer foraging (Matthews and Ferguson 2015). For Whale 1, which had T peaks only in the second half of the baleen plate, periodicity of T and degree of offset from nitrogen peaks was assessed using only the part of the plate that contained T peaks (more recently grown baleen).

Linear mixed effects models were used to test effects of age (fixed effect) on peak T concentration (dependent variable), with whale ID as a random effect to account for repeated measures within individual baleen plates. Change in baleen growth rate with age was also examined using a similar approach by modeling periods (time between peaks) of T cycles with age. Peak T concentrations and T periods were estimated for each complete hormone cycle, with a “T peak” defined as any single sample with T concentration higher than all four neighboring samples (the two prior and the two subsequent). Corresponding ages were calculated by subtracting the number of T cycles from the whale's estimated terminal age. Three candidate models—the null model, a random intercept model, and a random intercept and slope model—were fit separately to peak T concentrations and periods using maximum likelihood (ML) estimation using the R (version 4.0.5; R Core Team 2021) package nlme (Pinheiro et al. 2021). Optimal models were selected based on AIC corrected for small sample size (AICc), and refit using the restricted maximum likelihood (REML) method for parameter estimation (Zuur et al. 2009). Model fit and assumptions were assessed via visual inspection of residual plots for deviations from normality and homoscedasticity. Potential effects of whale age were additionally assessed across individuals via correlation of estimated terminal age (age at death) of each whale to the following metrics, each calculated as a single measure across the entire plate of each whale: mean T (across entire plate), median T (across entire plate), maximum T (across entire plate), baseline T (defined as mean of all T minima, each cycle having one T minimum), and %CV of T peaks; all these analyses had an *n* of nine (i.e., one data point for each whale), and were two-tailed with a significance threshold of 0.05. Analyses employed Prism 9 (www.graphpad.com) and base functions in R (R Core Team 2021).

## Results

### Regularly spaced T peaks

**Fig. 2:**
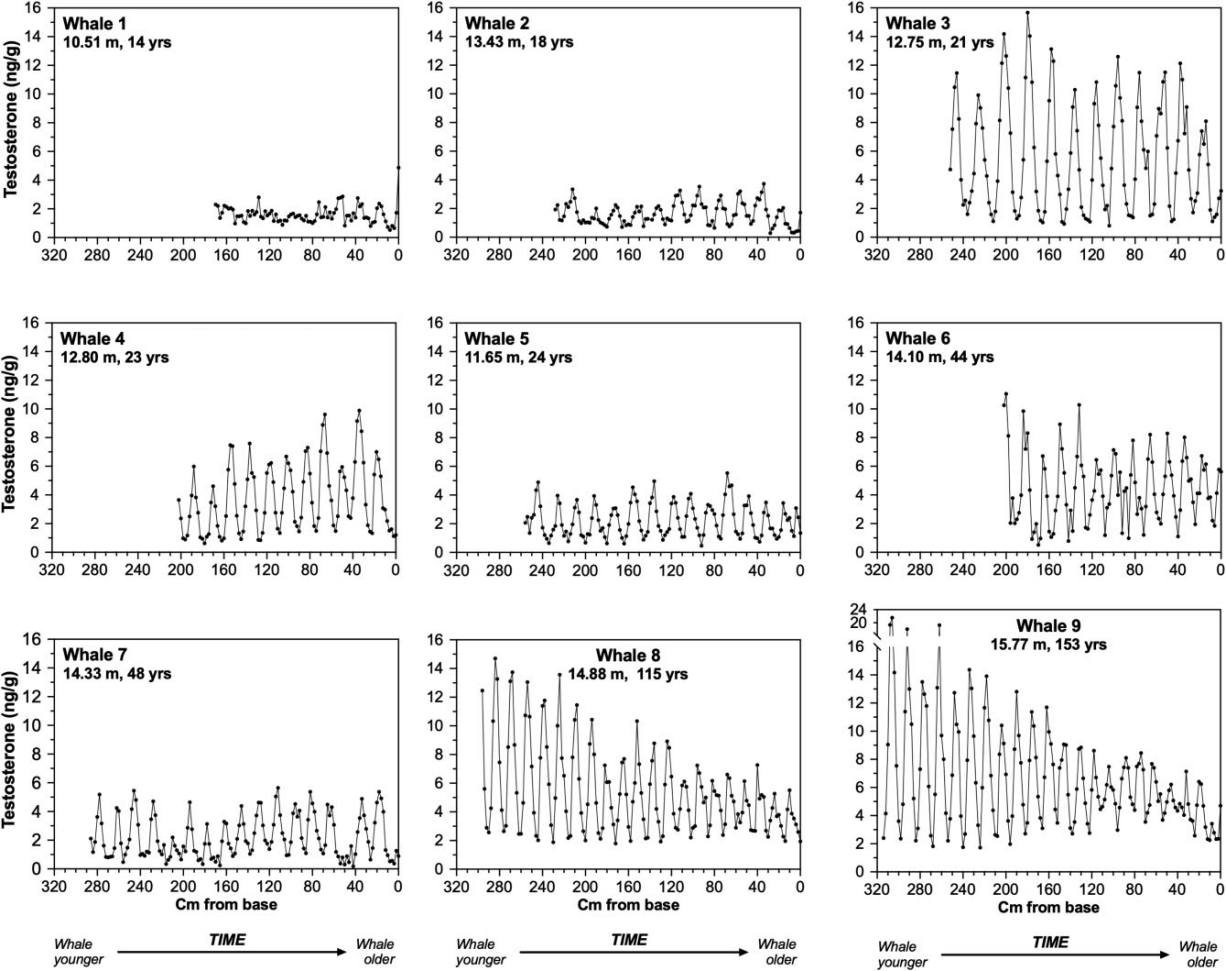
Longitudinal testosterone (T) concentrations at 2-cm increments along the full length of the longest baleen plate collected from each of nine male bowhead whales. Whales are numbered in order of estimated age (younger to older) with age estimated from aspartic acid racemization of the eye lens (preferred method) or body length (if eye lens not available). X-axis shows sampling location on the baleen plate, where 0 cm = most recently grown baleen (root of embedded plate for all whales except Whales 5 and 6; gumline for Whales 5 and 6). All graphs are shown to same scale to enable direct comparison of T peak spacing (on x-axis) and concentration (on y-axis); note Y-axis break of Whale 9 to accommodate unusually high T peaks.

Baleen of all nine whales contained regularly spaced regions of high T content (“T peaks”) separated by low-T (“baseline”) regions (Fig. 2, Table 2). In eight of nine whales, T peaks occurred at regularly spaced intervals (i.e., statistical analysis confirmed cyclicity with a predictable period for each whale) across the length of the baleen plate, with number of T peaks ranging from 11 to 22 (Fig. 2, Table 2). Spectral densities of high order autoregressive models fit to T profiles displayed single, pronounced narrow-band peaks with average individual periods ranging from 14.49–21.51 cm. The sole exception to the regular T cycling was Whale 1 (smallest whale, with youngest estimated age), which lacked T cycles in the first half of the baleen plate (baleen grown when the whale was younger), while the second half (baleen grown when the whale was older) contained five low-amplitude T peaks that were regularly spaced (Fig. 2).

**Table 2:** Characteristics of testosterone (T) cycles in baleen plates of nine adult male bowhead whales (*Balaena mysticetus*), quantified at 2 cm intervals for the complete length of each baleen plate. T concentrations are expressed in ng/g, nanograms of immunoreactive hormone per gram of baleen powder. Whales are ordered by estimated age (younger to older), from aspartic acid racemization of eye lens or body length.

Whale	Est. age (yrs)^a^	# T peaks	T cycle period (cm)	N cycle period^e^ (cm)	Phase relationship^f^ (months)	Mean T peak (ng/g) (± SD)	Mean T baseline^g^ (ng/g) (± SD)	Mean peak/baseline ratio^h^ (± SD)	Maximum T (ng/g)	Minimum T (ng/g)	Mean T (ng/g)	Median T (ng/g)
1	14	5^b^	18.35	15.50	0.00	3.06 ± 1.03	0.90 ± 0.29	4.04 ± 2.95	4.87	0.53	1.64	1.52
2	18	11	19.23	20.80	2.50	2.78 ± 0.66	0.73 ± 0.25	3.71 ± 1.37	3.73	0.28	1.65	1.49
3	21	12	21.51	20.60	3.35	11.77 ± 2.00	1.22 ± .28	9.70 ± 3.54	15.67	0.80	5.19	4.70
4	23	12	16.95	17.50	2.83	7.19 ± 1.50	1.22 ± 0.47	6.29 ± 1.74	9.89	0.62	3.80	3.14
5	24	15^c^	17.54	17.90	2.74	3.99 ± 0.72	0.81 ± 0.19	5.00 ± 1.35	5.53	0.46	2.23	1.91
6	44	13^c^	16.53	17.20	2.90	8.10 ± 1.59	1.36 ± 0.51	6.85 ± 3.40	11.05	0.51	4.39	4.05
7	48	16^d^	16.13	17.10	2.98	4.26 ± 1.22	0.72 ± 0.36	7.79 ± 5.82	5.65	0.18	2.21	1.76
8	115	20	14.49	14.60	3.31	9.10 ± 3.05	2.24 ± 0.32	4.12 ± 1.47	14.70	1.79	5.37	4.65
9	153	22	14.49	14.90	1.66	10.96 ± 4.53	2.83 ± 0.84	4.49 ± 2.77	21.56	1.72	6.61	5.82

a “Best available” age estimate, that is, from AAR if eye lens was available, or from body length if no eye lens was available.

b Baleen of Whale 1 had no T peaks for the first half of the baleen plate.

c Baleen of Whales 5 and 6 lacked the embedded root of the plate, which typically spans ∼1 year of baleen growth, *i.e.*, one T cycle may be missing (not included in # T peaks).

d Baleen of Whale 7 included fifteen peaks of similar amplitude plus one “short” T peak (included in total # of T peaks shown here), and a gap that may represent a “skipped” or “missing” T peak (not included here).

e N cycle period reprinted with permission for Whale 6 from Hunt et al. (2018); all other whales from Matthews & Ferguson (2015).

f Number of months by which T peak precedes N peak, assuming T and N cycles represent 12-mo years.

g Mean T baseline defined as mean of all T minima, each complete cycle having one minimum; partial cycles (at beginning or end of plate) not included.

h Mean T peak divided by mean T baseline.

### T periodicity and relationship to SI periodicity

**Fig. 3:**
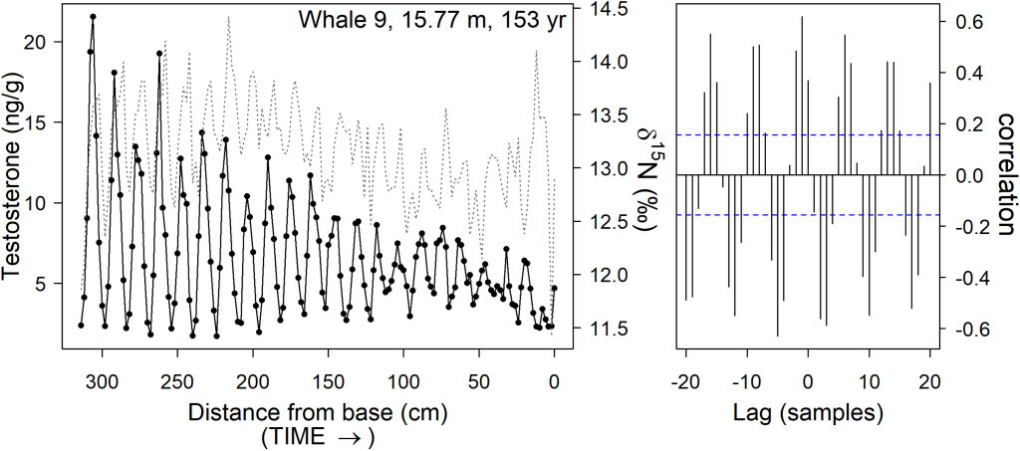
Comparison of testosterone (T) and stable nitrogen isotope (δ^15^N) patterns along a 330-cm baleen plate from a representative bowhead whale (Whale 9; see Supplementary Information for other whales). Left panel shows T concentrations (solid line) and δ^15^N (dotted line) measured at 2-cm increments along the length of the baleen plate. Note T peaks precede δ^15^N peaks, which is apparent in the cross-correlation function (right panel) as a high correlation at lag –1 (i.e., 2 cm) that repeats cyclically. Correlations falling outside dotted lines provide evidence of statistical significance at the 5% level. Stable isotope data reprinted from Matthews & Ferguson (2015) with permission.

Cross-correlation function (CCF) analyses indicated that T and δ^15^N periods were similar within each whale (Table 2, Fig. 3; see also Supplementary Information, Figs. S1–S8). In eight of nine whales, T peaks preceded δ^15^N peaks with a median phase offset of 2.8 ± 0.1 (SEM) months, assuming T and N periods both represent annual cycles (Whale 9 illustrated as an example in Fig. 3; for other whales see Supplementary Information, Figs S1–S8). T and δ^15^N cycles in the latter half of Whale 1’s baleen (smallest and youngest whale; Fig. S1) were synchronized (i.e. , no offset between the measures).

### Age-related changes in T cycles

**Fig. 4:**
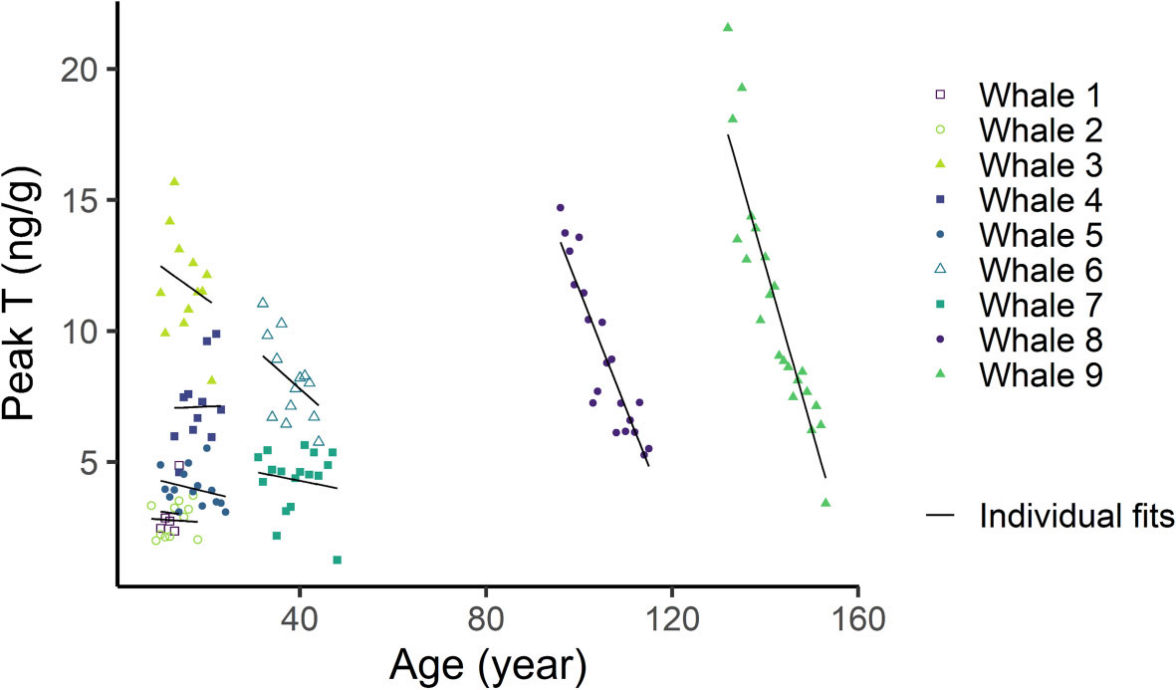
Individual fits (lines) estimated by a random intercept and slope linear mixed effects model fit to annual peak testosterone concentrations measured across baleen of nine different-aged male bowhead whales (see Table 3).

The random intercept and slope model was the optimal model of peak T concentration with age based on maximum likelihood estimation, with T peaks generally showing an overall decrease as a whale's age increased (–0.163 ± 0.082 ng g^–1^ year^–1^), albeit with considerable individual variation ranging from –0.625 to 0.008 ng g^–1^ year^–1^ (Table 3, Fig. 4). Notably, the two oldest whales exhibited much steeper declines in peak T concentration with age than the younger animals (Table 3,  Fig. 4). The random intercept model provided the best fit to T period, indicating T period decreased with whale age (–0.0445 ± 0.010 cm year^–1^) from an estimated overall intercept of 19.2 ± 0.67 cm (Table S1; Fig. S9). The same models are retained, with similar parameter estimates, when age is estimated by body length only (Tables S2 and S3, Figs S10 and S11).

**Table 3:** Candidate linear mixed effects models of peak testosterone concentrations measured across baleen of Eastern Canada-West Greenland male bowhead whales (*B. mysticetus*) and estimated age. The random intercept and slope model (bold), which was selected as the optimal model based on AICc values, indicates peak T (ng g^–1^) declines with age (year), with considerable variation in the rate of decline among individuals (see individual slope estimates for the fixed effect ‘age’).

Model	logLik	AICc			
*Null* Peak T ∼ 1, random = ∼1|whale	–309.2	624.6			
*Random intercept* Peak T ∼ age, random = ∼1|whale	–291.5	591.4			
***Random intercept and slope* Peak T ∼ age, random = ∼1+age|whale**	**–259.5**	**531.8**			
	**Estimate**	**SE**	**t-value**	**df**	**p-value**
**intercept**	**23.1**	**11.2**	**2.06**	**116**	**0.042**
**age**	**–0.163**	**0.082**	**–1.98**	**116**	**0.050**
	**intercept**	**age**			
Whale 1	**3.32**	**–0.0215**			
Whale 2	**2.93**	**–0.0118**			
Whale 3	**13.8**	**–0.128**			
Whale 4	**6.96**	**0.00804**			
Whale 5	**4.73**	**–0.0434**			
Whale 6	**14.1**	**–0.158**			
Whale 7	**5.69**	**–0.0351**			
Whale 8	**56.6**	**–0.450**			
Whale 9	**99.97**	**–0.625**			

Across individuals, median T (across entire plate), mean T (across entire plate), maximum T (single highest point on plate), minimum T (single lowest point on plate), and baseline T (average of all T minima within a whale) were all positively correlated with terminal age (*n* = 9 for all analyses; median T, *P* = 0.0267, *r^2^* = 0.5274; mean T, *P* = 0.0205, *r^2^* = 0.5591; maximum T, *P* = 0.0153, *r^2^* = 0.5925; minimum T, *P* = 0.0022, *r^2^* = 0.7604; baseline T, *P* = 0.0003, *r^2^* = 0.8680). T peaks throughout the plate also became more variable with whale age, with a significant positive correlation of age with the %CV of T peak concentration (*P* = 0.0188, *r^2^* = 0.5690). All comparisons remained significant, with the same directions of effect, when age was estimated by body length only (median T, *P* = 0.0341, *r^2^* = 0.4954; mean T, *P* = 0.0280, *r^2^* = 0.5216; maximum T, *P* = 0.0158, *r^2^* = 0.5886; minimum T, *P* = 0.0145, *r^2^* = 0.5981; baseline T, *P* = 0.0012, *r^2^* = 0.7983); and %CV of T peak concentration, *P* = 0.0206, *r^2^* = 0.5585).

## Discussion

Annual T cycles have long been theorized to occur in large whales since most species appear to be seasonal breeders, i.e., with defined calving seasons (∼1–3 months) coupled with predictable timing of annual migrations. Such life history traits in mammals typically co-occur with annual T cycles in males, with T peaks predictive of spermatogenesis and breeding. However, demonstration of T cycles in mysticete whales has proven elusive. Our data demonstrate occurrence of regularly spaced areas of high T concentration along baleen, with periods matching annual baleen growth rate (as estimated by SI analyses), indicating the regularly occurring T periods are annual cycles. The negative relationship of T period with whale age agrees with the known slowing of baleen growth rate as whales age, providing additional confirmation that the T periods are annual cycles (Lubetkin et al. 2008; Lubetkin et al. 2012). Assuming baleen hormone content reflects circulating plasma concentrations when the baleen was grown—an interpretation supported by progesterone and glucocorticoid data in this and other species (Hunt et al. 2016, 2017a, 2017b, 2018; Rolland et al. 2017; Lysiak et al. 2018; Fernández Ajó et al. 2020; Lowe et al. 2021)—our data indicate that individual male bowheads routinely experience predictable annual T cycles for decades of adult life.

Bowhead whales have unusually large testes relative to body size, and are thought to employ a scramble-competition mating system involving multiple males jockeying for position around a single female, a behavior also observed in their close relatives the right whales, *Eubalaena spp.* (Frasier et al. 2007; MacLeod 2010; Würsig and Koski 2021). These traits suggest sperm competition, which in mammals typically entails dramatic seasonal T peaks that elevate coincident with courtship behavior, insemination, and fertilization (Dixson and Anderson 2004; Frasier et al. 2007). Some mammals, however, can and do decouple some of these reproductive events via mechanisms such as sperm storage and/or embryonic diapause (Birkhead and Møller 1993; Orr and Zuk 2014; Holt and Fazeli 2015). Though we cannot rule out such phenomena in bowhead whales, consistent differences in length of fetuses in pregnant female bowhead whales harvested in spring vs. fall months and a restricted calving season from April to early June imply a single seasonal mating period, and are inconsistent with diapause or sperm storage being major features of bowhead whale reproduction (Nerini et al. 1984; Tarpley et al. 2021; Würsig and Koski 2021). Thus, we assume that T peaks are coincident with seasonal mating and fertilization. Eight of nine males in this study had T peaks preceding putative summer δ^15^N peaks by approximately three months, suggesting that breeding in this bowhead whale population occurs during late winter to spring, consistent with observations of increased social activity among bowhead whales from March through May (Tervo et al. 2009, 2011; Würsig and Koski 2021). Satellite telemetry data show that the majority of this bowhead whale population (East Canada-West Greenland population, EC-WG) is located in Hudson Strait and off southeastern Baffin Island at this time (Ferguson et al. 2010; Fortune et al. 2020; Fig. 1), while some large mature females congregate in Disko Bay (Heide-Jørgensen et al. 2010). These regions may therefore represent important mating habitats. This illustrates the potential utility of longitudinal hormone data for assessing the likely location of mating grounds and, thus, informing population management decisions (Laidre et al. 2008; Kovacs et al. 2011).

The relationships noted here between peak T concentrations and estimated whale age suggest potential influences of maturation and senescence. Firstly, the T patterns seen in young whales suggest that T cycling may begin at a considerably younger age than expected. The age of sexual maturity in male bowheads has been estimated in several studies as ∼20–25 years, based on a significant increase in mean testis size that begins at approximately 12.5 m body length (George et al. 2021). In this study, the youngest whale (Whale 1, 10.51 m body length) lacked T cycles in the first half of his plate, followed by five low-amplitude, but regularly spaced, annual T cycles in the second half of the plate. Given this whale's estimated age-at-death (from AAR) of 14 yr, his T profile thus suggests that his first T cycle occurred at the age of 9 yr. Using a similar approach of subtracting number of documented T cycles from estimated age at death, Whales 2 through 5 had likely been experiencing T cycles since at least the ages of 7, 9, 11, and 9 yr, respectively. For Whales 1, 2, 3, and 5, these estimates rely on accuracy of the AAR eye lens age estimates. Standard errors of AAR-derived age estimates of young (i.e., age 20–30 years) bowhead whales range from ∼6–9 yr (George et al. 1999), but even assuming the extreme of a 9-year underestimate of true age, Whales 1, 2, 3, and 5 all began T cycling before the age of 20 yr, as did Whale 4 (age estimated only by body length). Thus, our data suggest that young male bowhead whales begin seasonal T cycles well in advance of the increase in testis mass that is customarily used to estimate onset of sexual maturity. We note, however, that the first seasonal T cycles in young male mammals do not necessarily indicate full reproductive competence; body size, social dominance, experience, male-male competition, and female choice, as well as other factors, can separate age of first T cycling from age of first paternity by many years (Harcourt et al. 2006; Frasier et al. 2007).

At the other extreme, T patterns in older males showed complex relationships with age. Across whales, T peaks generally reached higher concentrations in older whales, for example, with both median T (across entire plate) and maximum T (single highest point) highest in the oldest whales. However, within a given whale's baleen plate, that whale's T peaks generally tended to decline over successive years. These declines in T peak concentration were an order of magnitude greater in older whales than in the younger whales (some of which showed increasing peak T concentrations with age; Figs. 2, 4, and S9). It is possible that variation in extraction efficiency in older baleen (tip of plate) compared to younger baleen (base of plate) might result in an apparent decline of T over successive years that does not accurately represent circulating plasma concentrations. However, the markedly steeper declines in peak T concentration in the oldest whales were accompanied by a simultaneous increase in baseline T (Fig. 2). This increase in baseline T, despite a simultaneous decrease in T peaks across time, is not concordant with an extraction-efficiency effect. Though the very small sample size of older males (*n* = 2 whales over 50 yr of age) prevents firm conclusions at present, it is possible that these patterns seen in the two oldest males might represent influences of reproductive senescence. If so, the increase in baseline T could represent a generalized loss of cyclicity with increasing age. The oldest individual, Whale 9, showed some other indications of loss of cyclicity; his last five cycles became progressively less regular in amplitude and period, with his very last T peak so small that it may not have been physiologically relevant (Fig. 3). Notably, bowheads have the longest lifespan of any known mammal, >200 yr (George and Bockstoce 2008; George et al. 2021). Though a greater sample size of older males is clearly needed, together the T profiles of Whales 8 and 9 suggest that though bowhead whales are known for attaining great age, the oldest male individuals may no longer be fully reproductively competent. Prior studies have reported two old bowhead males (>100 years by body length) to have anomalous testicular morphology, while a female >130 yr had much lower corpora lutea counts than expected (Tarpley et al. 1995; George et al. 2011). Generally, species that employ intense sperm competition are often characterized by increased reproductive senescence in males, particularly in those individuals that had highest reproductive success earlier in life (Lemaître et al. 2020). Thus, the sperm-competition strategy that is apparently employed by male bowheads and other *Balaenidae* may be linked to occurrence of reproductive senescence in older males late in life.

If the steep declines in T peaks in the two oldest individuals do represent senescence, the cause may be endogenous, i.e., loss of hypothalamic or pituitary capacity for initiating regular annual T cycles or loss of testicular steroidogenic capability. Social factors can also influence T concentrations; in many vertebrates, T elevates not just coincident with, but in response to, male-male competition as well as interactions with females (Kempenaers et al. 2008; Wingfield et al. 2020). Thus, reduced competitiveness, and/or a behavioral choice not to compete or not to migrate to breeding grounds, may in and of itself cause or exacerbate declines in T in the oldest individuals. An additional possibility is accumulation of endocrine-disrupting toxicants over time; though bowheads are generally assumed to be less vulnerable to such effects than other cetaceans due to their relatively low trophic level (i.e., feeding primarily on zooplankton), their very long lifespan may eventually allow such pollutants to reach tissue concentrations that have physiological impacts (Hoekstra et al. 2002; Schultz et al. 2021). Further data will be necessary to explore whether the progressive decline in T peaks noted in the (admittedly small) dataset presented here is a common pattern in male bowheads, and if so, whether it is primarily due to intrinsic vs. extrinsic factors, and whether it influences probability of paternity in ways that could be incorporated into population models.

Some of the above patterns may be artefacts of small sample size or of methodology (e.g., age estimation errors), particularly as not all age classes are equally represented in the sample set. These unavoidable limitations in study design reflect logistical constraints of studying mysticete whales. Baleen cannot be collected from living whales, and as stranded bowhead carcasses are exceedingly rare and logistically challenging to necropsy, samples are limited to baleen collected from indigenous hunts; but few bowheads are harvested per year and large adults are rarely harvested (Ferguson et al. 2021). Thus, sample size and age structure must necessarily be opportunistic. Furthermore, it is not possible to compare baleen data to longitudinal plasma hormone profiles, as there is no method for live-capture or for blood sampling from free-swimming large whales. Additionally, age estimation methods are known to be imperfect (e.g., body length in closely related species can be influenced by nutrition and stress; Stewart et al. 2021; see also George et al., 2021). Details of hormone deposition into baleen, possible alterations in extraction efficiency in older baleen, and the exact chemical identity of the compounds identified by the hormone assays, also remain to be confirmed (though excellent assay parallelism strongly suggests presence of testosterone or a closely related androgen; Hunt et al. 2017b, 2018). Thus, our study cannot provide definitive answers as to the potential role of age in bowhead reproduction, but our data do suggest an intriguing potential relationship that we recommend be further explored in future studies. As the field of mysticete baleen research continues to grow, we encourage researchers and especially necropsy field crews to routinely gather those tissues that can be used not just for longitudinal hormone analyses (baleen, earwax plug) but also for age estimation (e.g., eye lens, potentially tissues for epigenetic analyses, length of the longest piece of baleen, detailed information on morphological features, etc.; George et al., 2021).

Finally, several whales in this study exhibited intra-individual or inter-individual variation in T that remains unexplained. For example, Whale 7 had two anomalously “short” T peaks in an otherwise regular sequence of T peaks, an example of unexplained inter-annual variation within an individual (Fig. 2). Unexplained variation between individuals occurred as well; for example, Whale 3 had relatively high T peaks throughout his plate despite a relatively young estimated age, and Whale 7 had relatively low T peaks throughout his plate despite a relatively old estimated age (Fig. 2). While some of the latter findings may be due to inaccuracies in age estimates, it is well-established in mammalian endocrinology that multiple factors besides age can and typically do modulate T levels. Such factors often include body condition, stress, a suite of numerous social effects (e.g., presence of females, success in gaining access to females, social cues from females, interactions with other males, experience, dominance), as well as individual personality differences, epigenetic effects, immune costs, alternative mating strategies, and more (Reeder and Kramer 2005; Greiner et al. 2010; Muehlenbein and Watts 2010; O'Brien et al. 2018; Ball and Balthazart 2020; Kraus et al. 2020; Moore et al. 2020; Dalle Luche et al. 2021; Sugianto et al. 2021; Tomiyasu et al. 2021). Many of these influences on T involve mechanisms that are evolutionarily conserved across vertebrates (e.g., direct effects of thyroid hormones and glucocorticoids on hypothalamic and pituitary release of reproductive hormones; Martin et al. 2008; Singh et al. 2011; Oyola and Handa 2017). Thus, we regard it as likely that many of these factors may be at play in mysticete whales as well. Some existing data do suggest potential effects of body condition or stress on patterns of T concentration; for example, blubber T in humpback whales (*Megaptera novaeangliae*) was recently found to be negatively correlated with blubber adiposity (Dalle Luche et al. 2021). In North Atlantic right whales, one case has been reported of a “small” T peak in baleen of a single male whale in one year that was preceded by a large elevation in glucocorticoids in the prior year (Hunt et al. 2018); this case, while anecdotal, suggests potential effects of stress on male reproduction. These possible modulatory influences on T in individual male whales remain largely unexplored, but baleen analysis offers the potential to address these questions in future analyses that could assess not only T but also glucocorticoids, thyroid hormones, and other relevant metrics such as blubber thickness, body condition, and potentially sightings records and behavioral data.

In conclusion, our retrospective analysis of T patterns along baleen of male bowhead whales confirms the occurrence of regular annual T cycles consistent with seasonal mating, with timing of peak T concentrations corresponding with known mating in late winter / early spring. Our data additionally suggest that this longest-lived mammalian species may reach physiological maturity (i.e., activation of the hypothalamo-pituitary-gonadal axis and onset of T cycling) earlier than thought, and that reproductive senescence may occur in the oldest individuals. In other whale species and populations, study of male T cycles could similarly help clarify season of mating and could also, in combination with telemetry or sightings data, help determine or confirm likely location of mating. We encourage continued analysis of baleen from stranded and harvested whales and exploration of baleen archives that exist in many museums, so as to further investigate patterns of reproductive seasonality, sexual maturity, and influences of age and life stage in this and other species, in both present-day and past populations. Ultimately, the analysis of multi-decade longitudinal endocrine data may not only illuminate questions of basic interest regarding reproductive biology, but could also be of utility for population management of bowhead whales in the changing Arctic, and of other large whales recovering from over-exploitation during commercial whaling and coping with ongoing anthropogenic changes to their environment.

## Supplementary Material

obac014_Supplemental_File
