# Supplementary material for: Male Bowhead Whale Reproductive Histories Inferred from Baleen Testosterone and Stable Isotopes
Source: Integr Org Biol. 2022 May 4;4(1):obac014. doi: 10.1093/iob/obac014 (PMC9125798; doi:10.1093/iob/obac014)
Supplement: obac014_Supplemental_File [file obac014_supplemental_file.docx]

**SUPPLEMENTARY INFORMATION**


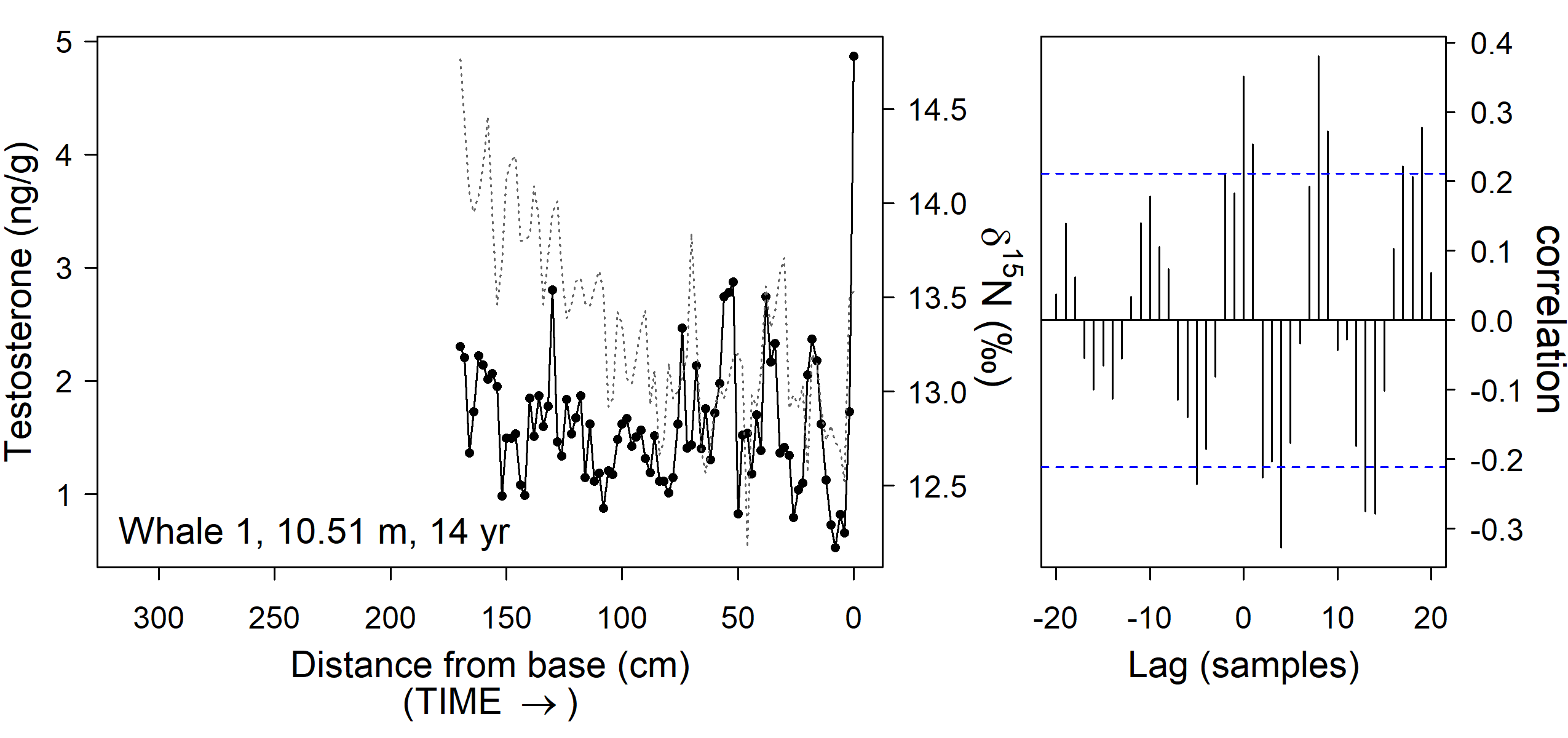


**Figure S1.** Comparison of testosterone (T) and stable nitrogen isotope (δ^15^N) patterns along a 181-cm baleen plate from Whale 1. Left panel shows T concentrations (solid line) and δ^15^N (dotted line) measured at 2-cm increments along the length of the baleen plate. Unlike all the other whales in the sample, in which T peaks preceded δ^15^N peaks by 1-3 samples, T and δ^15^N in Whale 1 cycled in-phase, with the highest correlation occurring at lag 0 (see cross-correlation function; right panel). This pattern persisted when both time series were restricted to the newest 80 cm of baleen growth where T cycles are visibly discerned. Correlations falling outside dotted lines provide evidence of statistical significance at the 5% level. Stable isotope data reprinted from Matthews & Ferguson (2015) with permission.


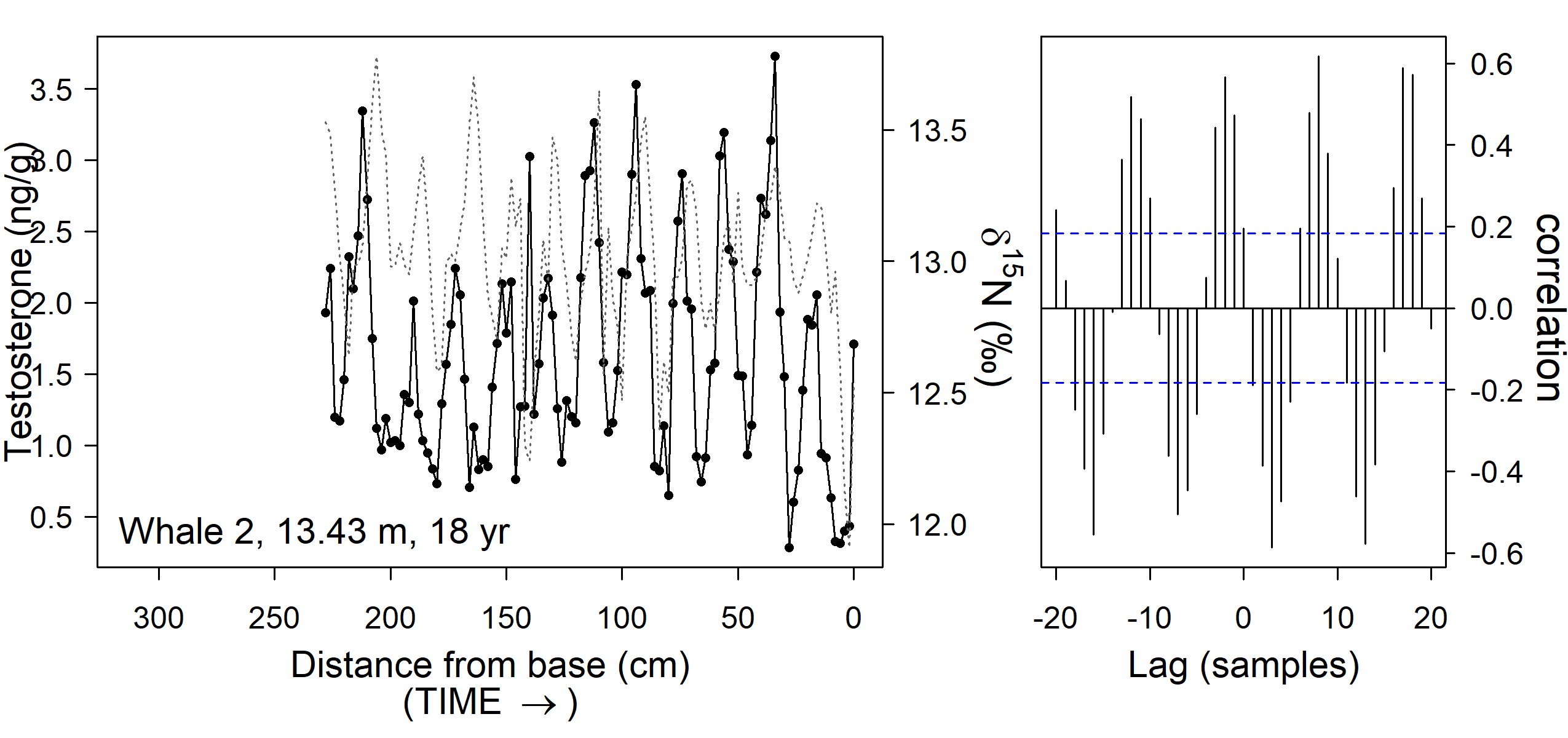


**Figure S2.** Comparison of testosterone (T) and stable nitrogen isotope (δ^15^N) patterns along a 235-cm baleen plate from Whale 2. Left panel shows T concentrations (solid line) and δ^15^N (dotted line) measured at 2-cm increments along the length of the baleen plate. Note T peaks precede δ^15^N peaks, which is apparent in the cross-correlation function (right panel) as a high correlation at lag –2 (i.e., 4 cm) that repeats cyclically. Correlations falling outside dotted lines provide evidence of statistical significance at the 5% level. Stable isotope data are reprinted from Matthews & Ferguson (2015) with permission.


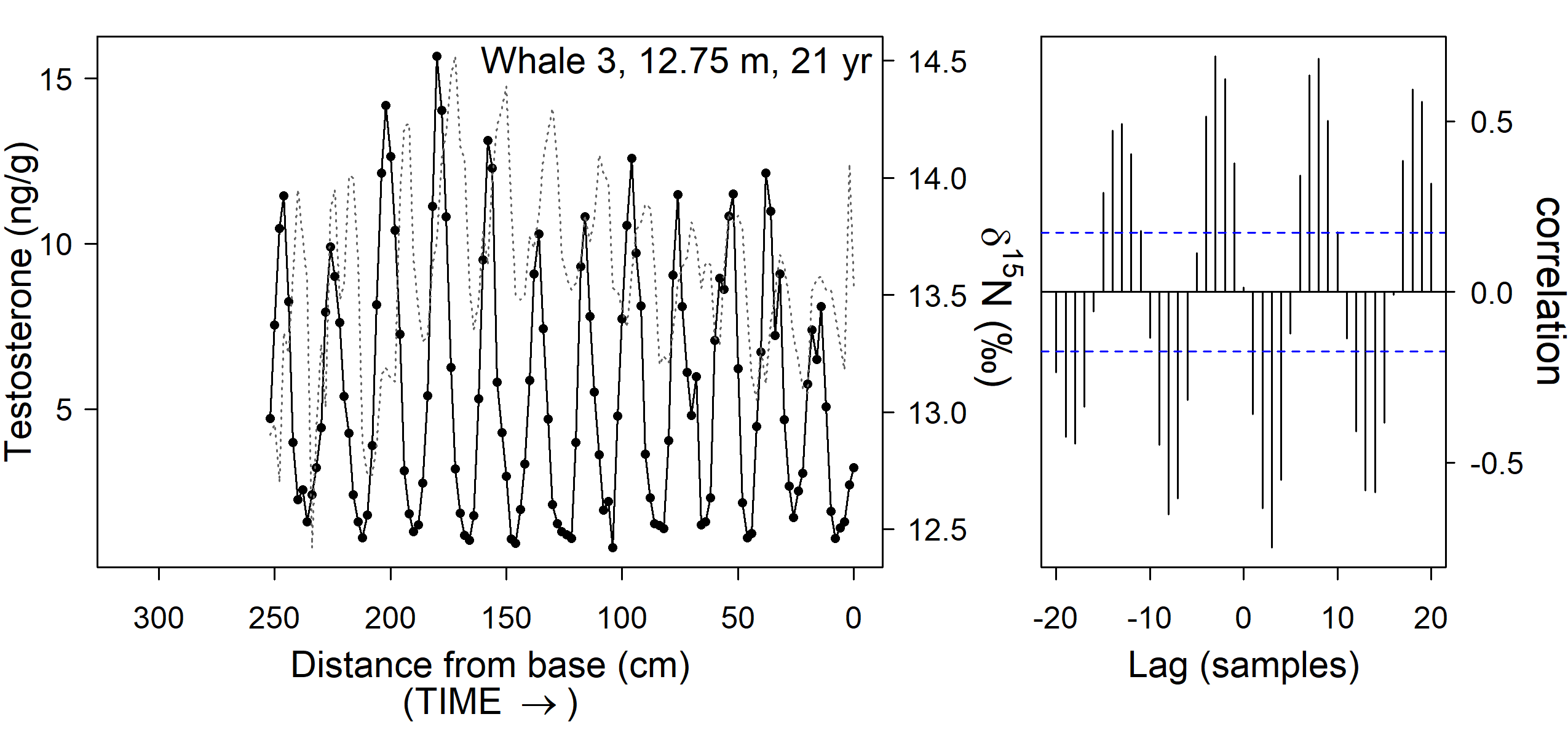


**Figure S3.** Comparison of testosterone (T) and stable nitrogen isotope (δ^15^N) patterns along a 266-cm baleen plate from Whale 3. Left panel shows T concentrations (solid line) and δ^15^N (dotted line) measured at 2-cm increments along the length of the baleen plate. Note T peaks precede δ^15^N peaks, which is apparent in the cross-correlation function (right panel) as a high correlation at lag –3 (i.e., 6 cm) that repeats cyclically. Correlations falling outside dotted lines provide evidence of statistical significance at the 5% level. Stable isotope data are reprinted from Matthews & Ferguson (2015) with permission.


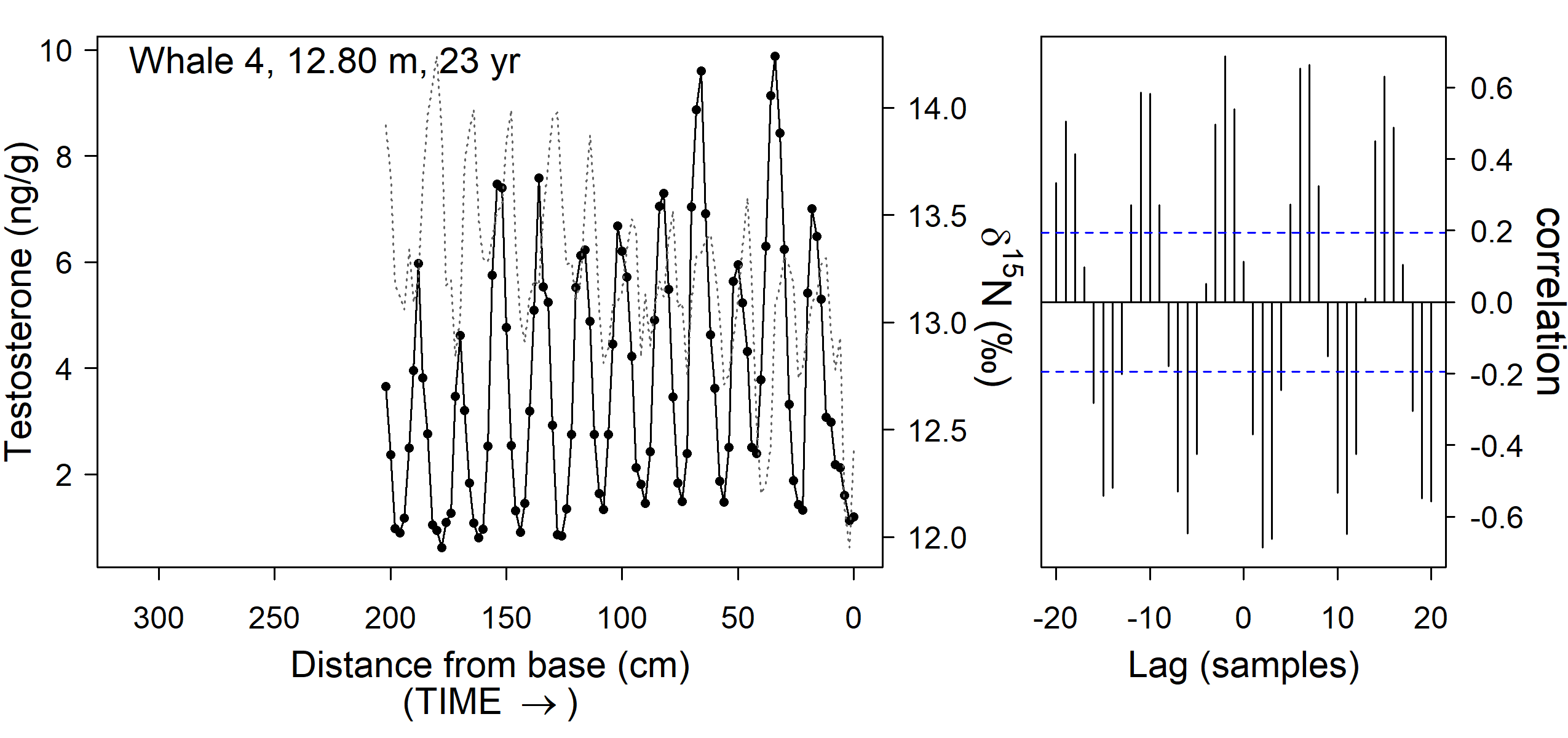


**Figure S4.** Comparison of testosterone (T) and stable nitrogen isotope (δ^15^N) patterns along a 230-cm baleen plate from Whale 4. Left panel shows T concentrations (solid line) and δ^15^N (dotted line) measured at 2-cm increments along the length of the baleen plate. Note T peaks precede δ^15^N peaks, which is apparent in the cross-correlation function (right panel) as a high correlation at lag –2 (i.e., 4 cm) that repeats cyclically. Correlations falling outside dotted lines provide evidence of statistical significance at the 5% level. Stable isotope data are reprinted from Matthews & Ferguson (2015) with permission.


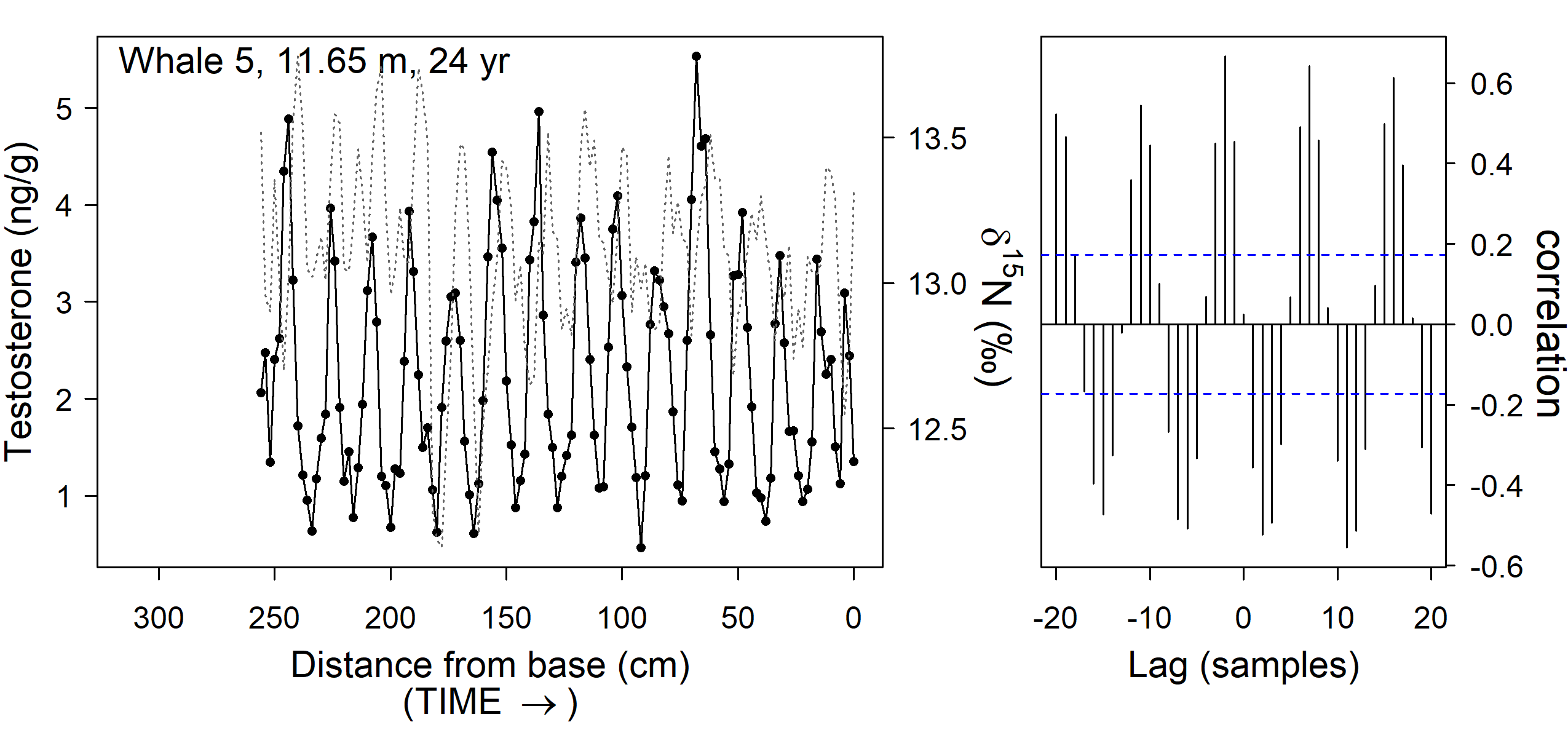


**Figure S5.** Comparison of testosterone (T) and stable nitrogen isotope (δ^15^N, from [19]) patterns along a 270-cm baleen plate from Whale 5. Left panel shows T concentrations (solid line) and δ^15^N (dotted line) measured at 2-cm increments along the length of the baleen plate. Note T peaks precede δ^15^N peaks, which is apparent in the cross-correlation function (right panel) as a high correlation at lag –2 (i.e., 4 cm) that repeats cyclically. Correlations falling outside dotted lines provide evidence of statistical significance at the 5% level. Stable isotope data are reprinted from Matthews & Ferguson (2015) with permission.


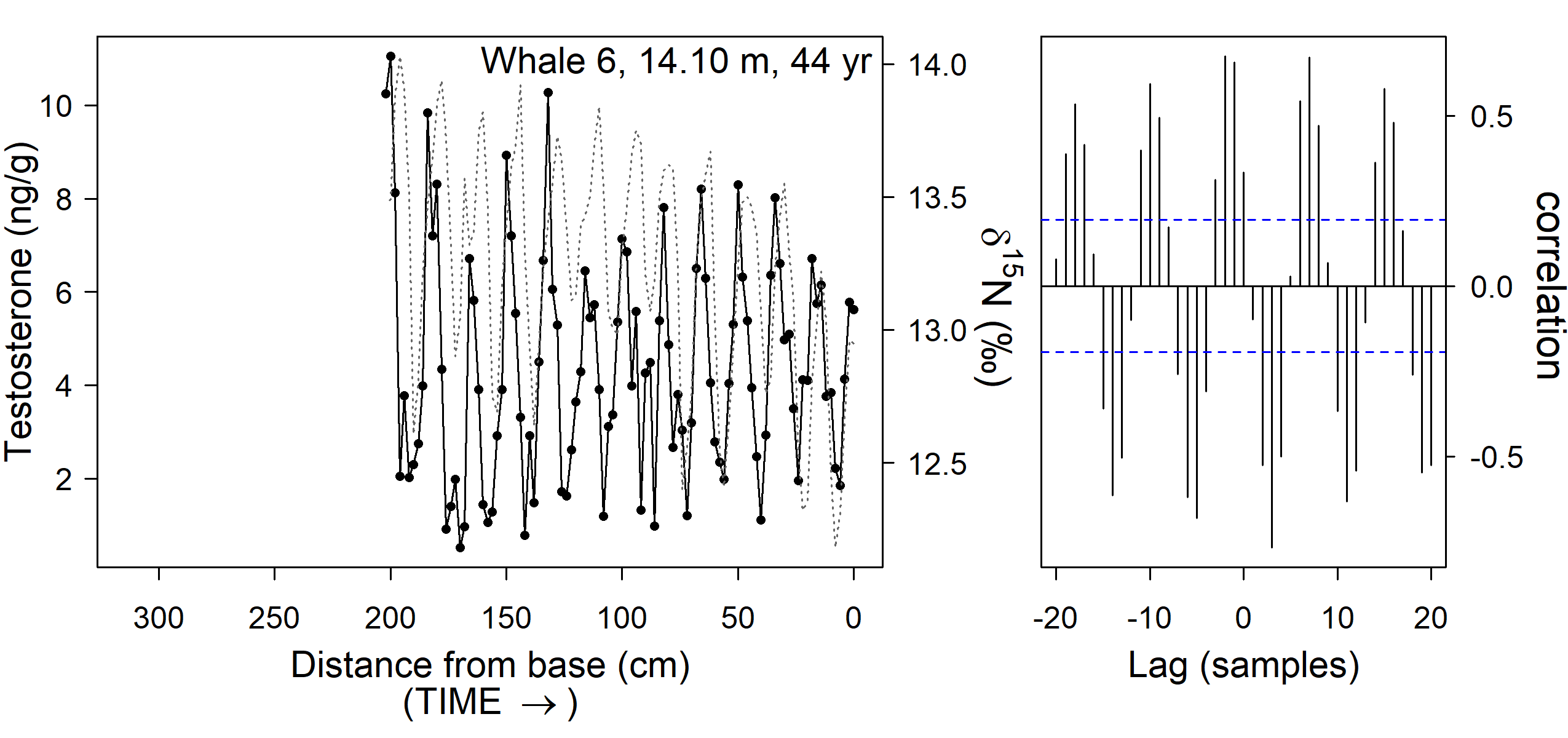


**Figure S6.** Comparison of testosterone (T) and stable nitrogen isotope (δ^15^N) patterns along a 204-cm baleen plate from Whale 6. Left panel shows T concentrations (solid line) and δ^15^N (dotted line) measured at 2-cm increments along the length of the baleen plate. Note T peaks precede δ^15^N peaks, which is apparent in the cross-correlation function (right panel) as a high correlation at lag –2 (i.e., 4 cm) that repeats cyclically. Correlations falling outside dotted lines provide evidence of statistical significance at the 5% level. Hormone and stable isotope data reprinted from Matthews & Ferguson (2015) with permission.


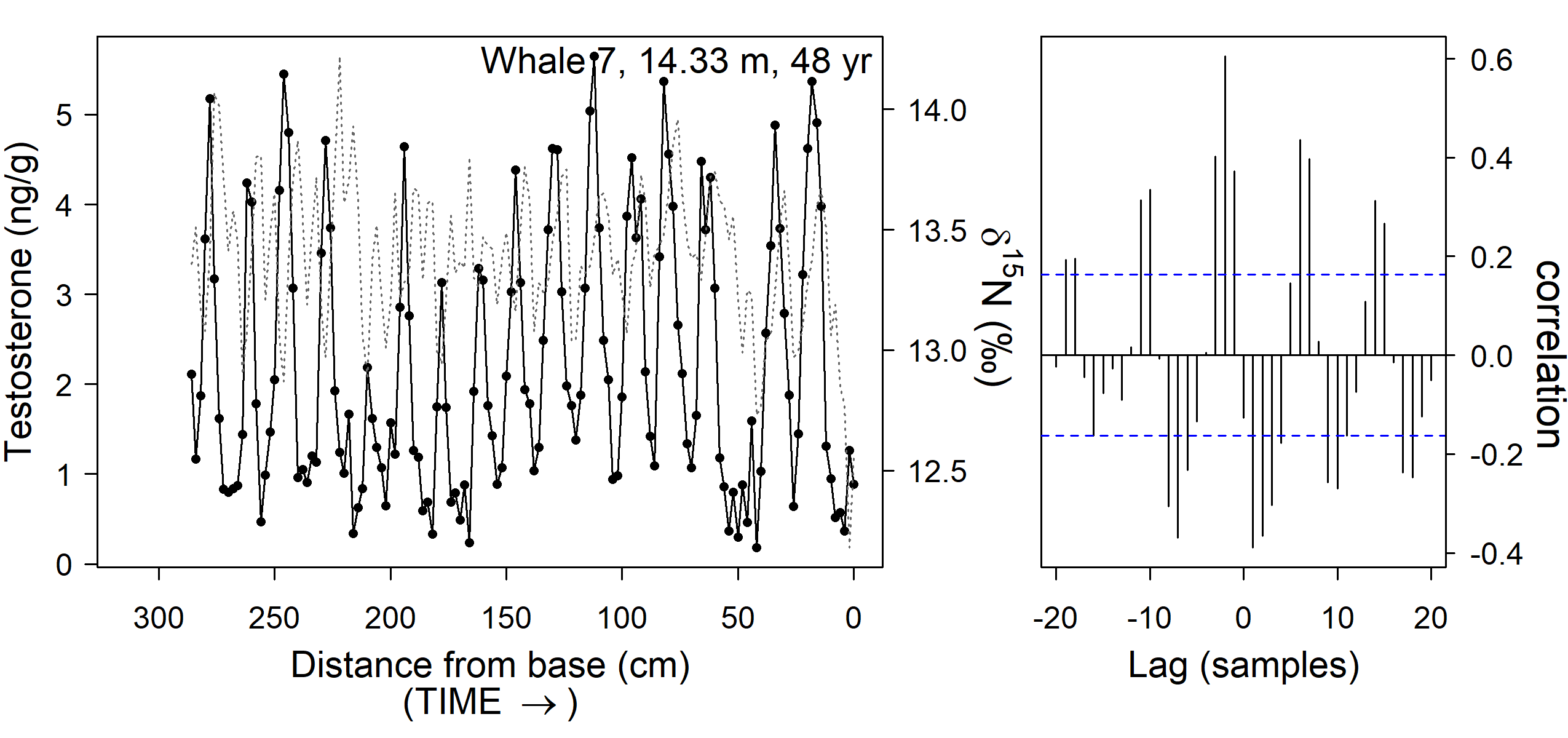


**Figure S7.** Comparison of testosterone (T) and stable nitrogen isotope (δ^15^N) patterns along a 298-cm baleen plate from Whale 7. Left panel shows T concentrations (solid line) and δ^15^N (dotted line) measured at 2-cm increments along the length of the baleen plate. Note T peaks precede δ^15^N peaks, which is apparent in the cross-correlation function (right panel) as a high correlation at lag –2 (i.e., 4 cm) that repeats cyclically. Correlations falling outside dotted lines provide evidence of statistical significance at the 5% level. Stable isotope data are reprinted from Matthews & Ferguson (2015) with permission.


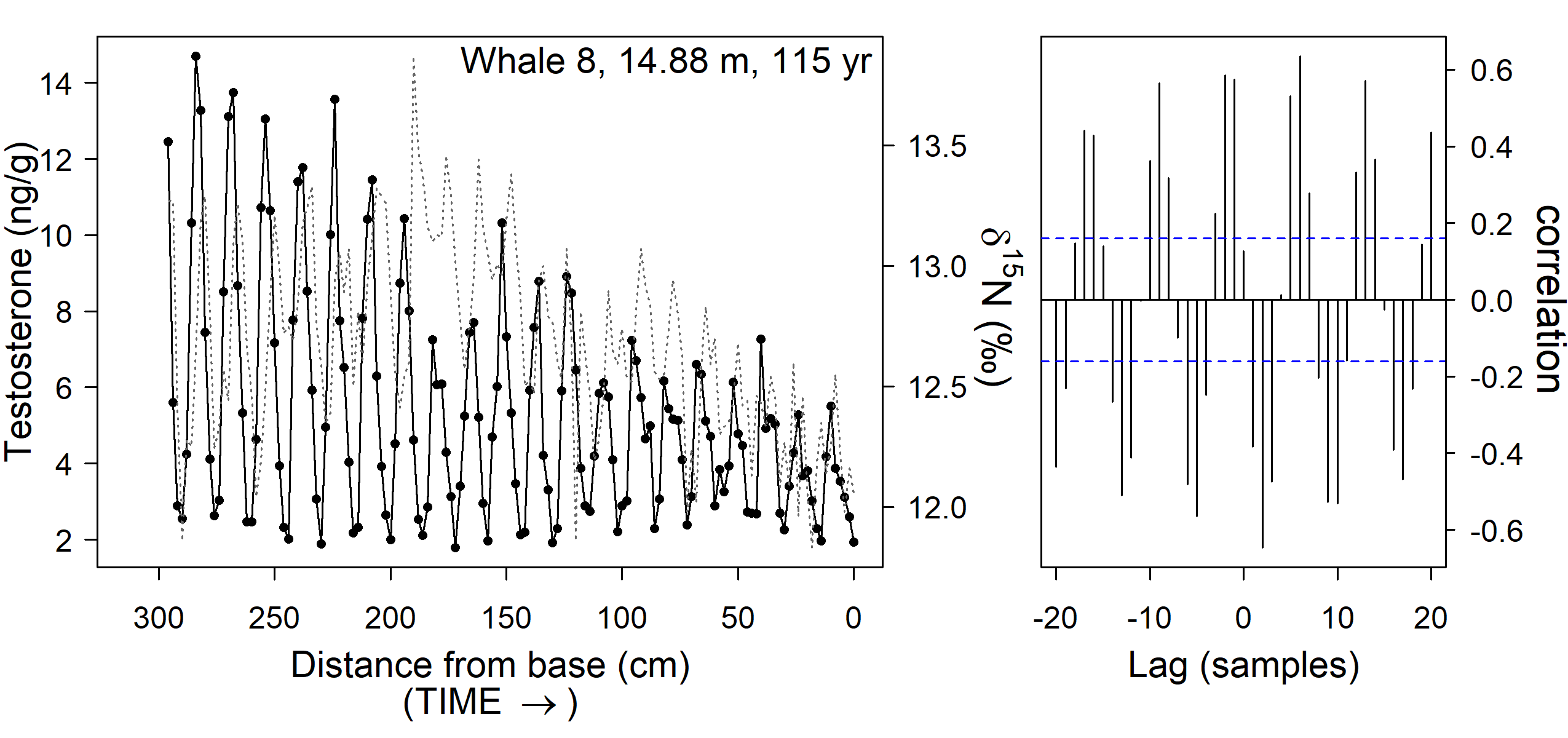


**Figure S8.** Comparison of testosterone (T) and stable nitrogen isotope (δ^15^N) patterns along a 319-cm baleen plate from Whale 8. Left panel shows T concentrations (solid line) and δ^15^N (dotted line) measured at 2-cm increments along the length of the baleen plate. Note T peaks precede δ^15^N peaks, which is apparent in the cross-correlation function (right panel) as a high correlation at lag –2 (i.e., 4 cm) that repeats cyclically. Correlations falling outside dotted lines provide evidence of statistical significance at the 5% level. Stable isotope data are reprinted from Matthews & Ferguson (2015) with permission.


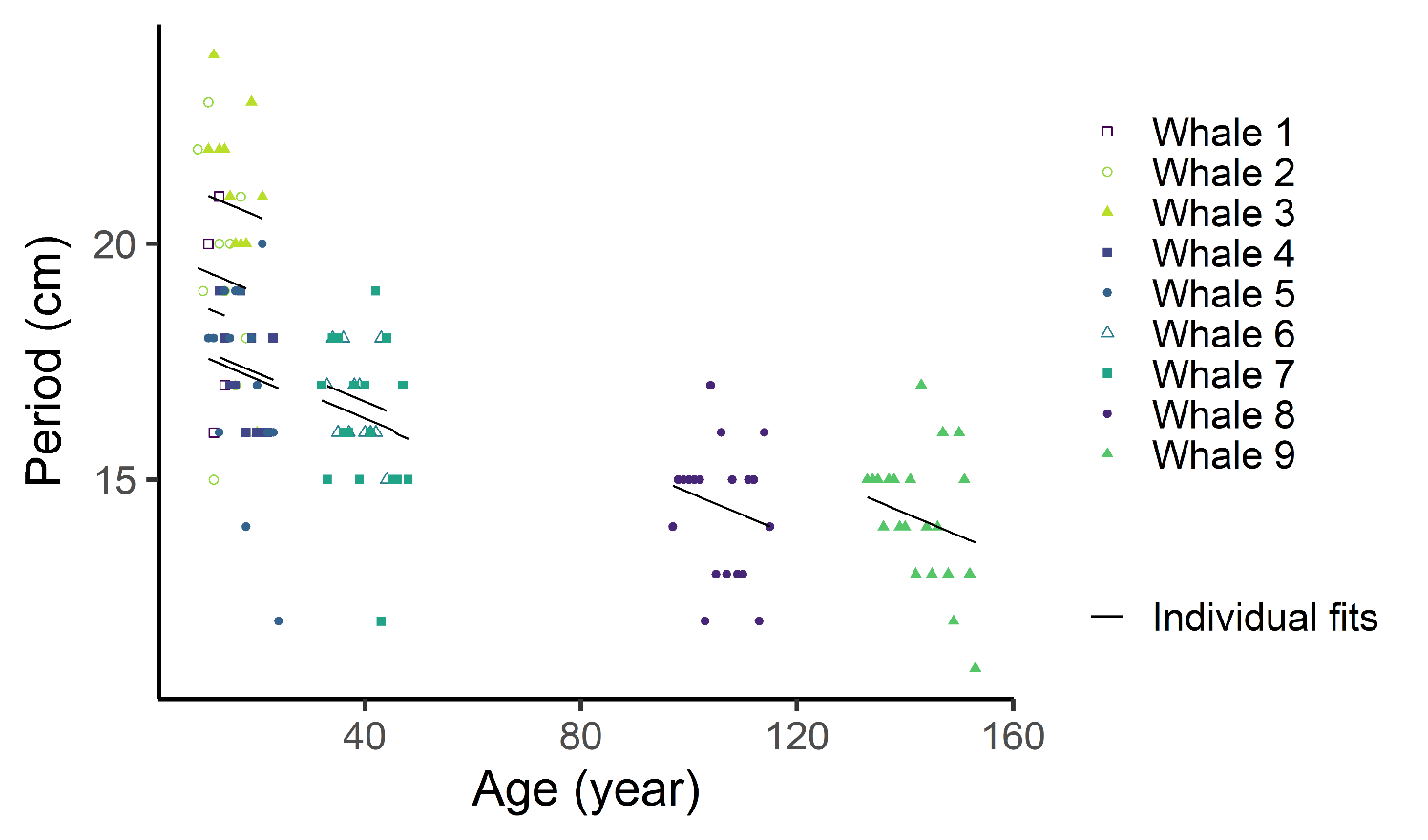


**Figure S9.** Individual fits (lines) estimated by a random intercept linear mixed effects model fit to periods of annual testosterone cycles across baleen of nine different-aged male bowhead whales (see Table S1).

**
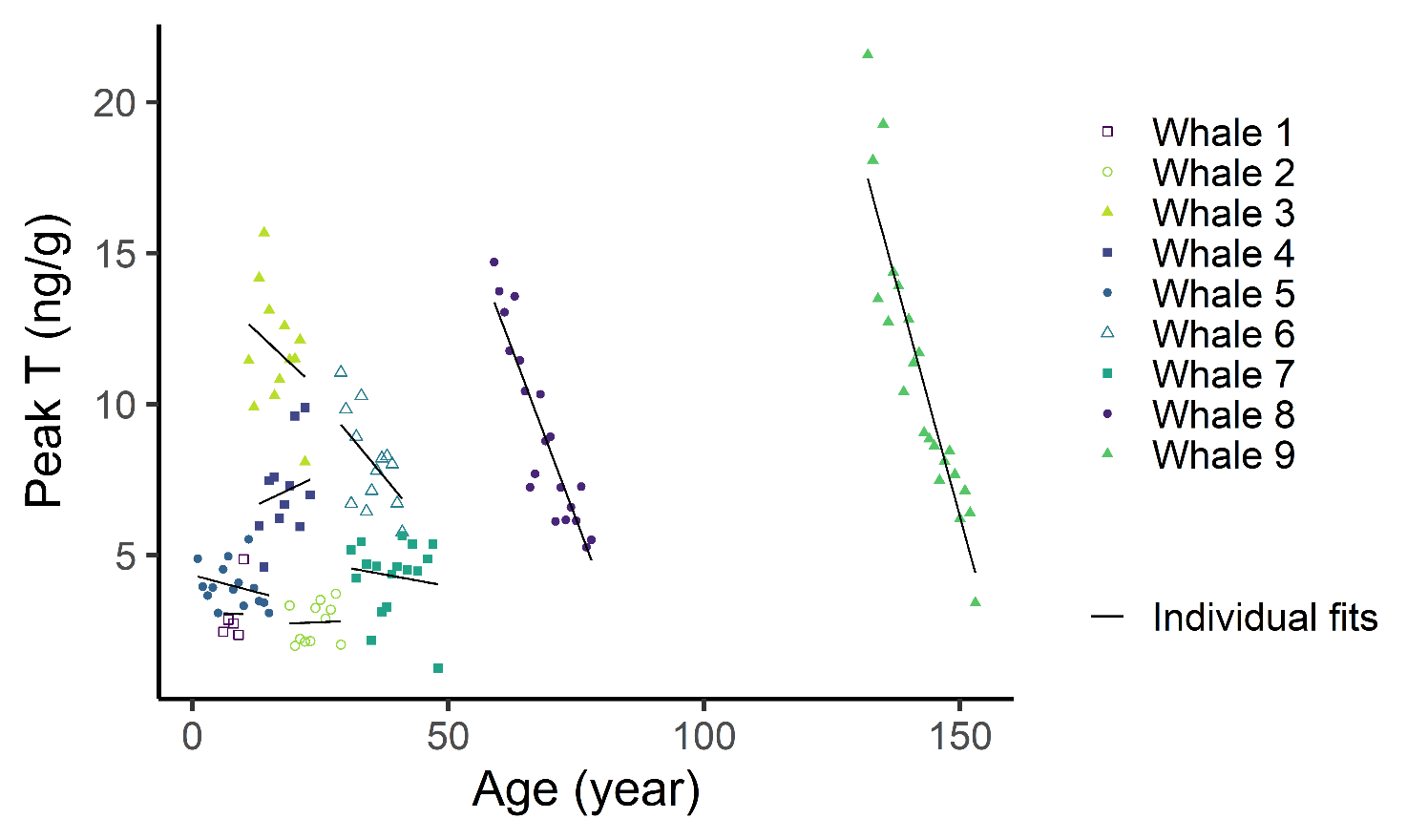
**

**Figure S10:** Individual fits (lines) estimated by a random intercept and slope linear mixed effects model fit to annual peak testosterone concentrations measured across baleen of nine different-aged male bowhead whales. This model was fit to ages estimated using body length-at-age relationships only, in comparison to the same model fit to ages estimated using a combination of aspartic acid racemization (AAR) and length-at-age relationships (see Figure 4; Table S2).


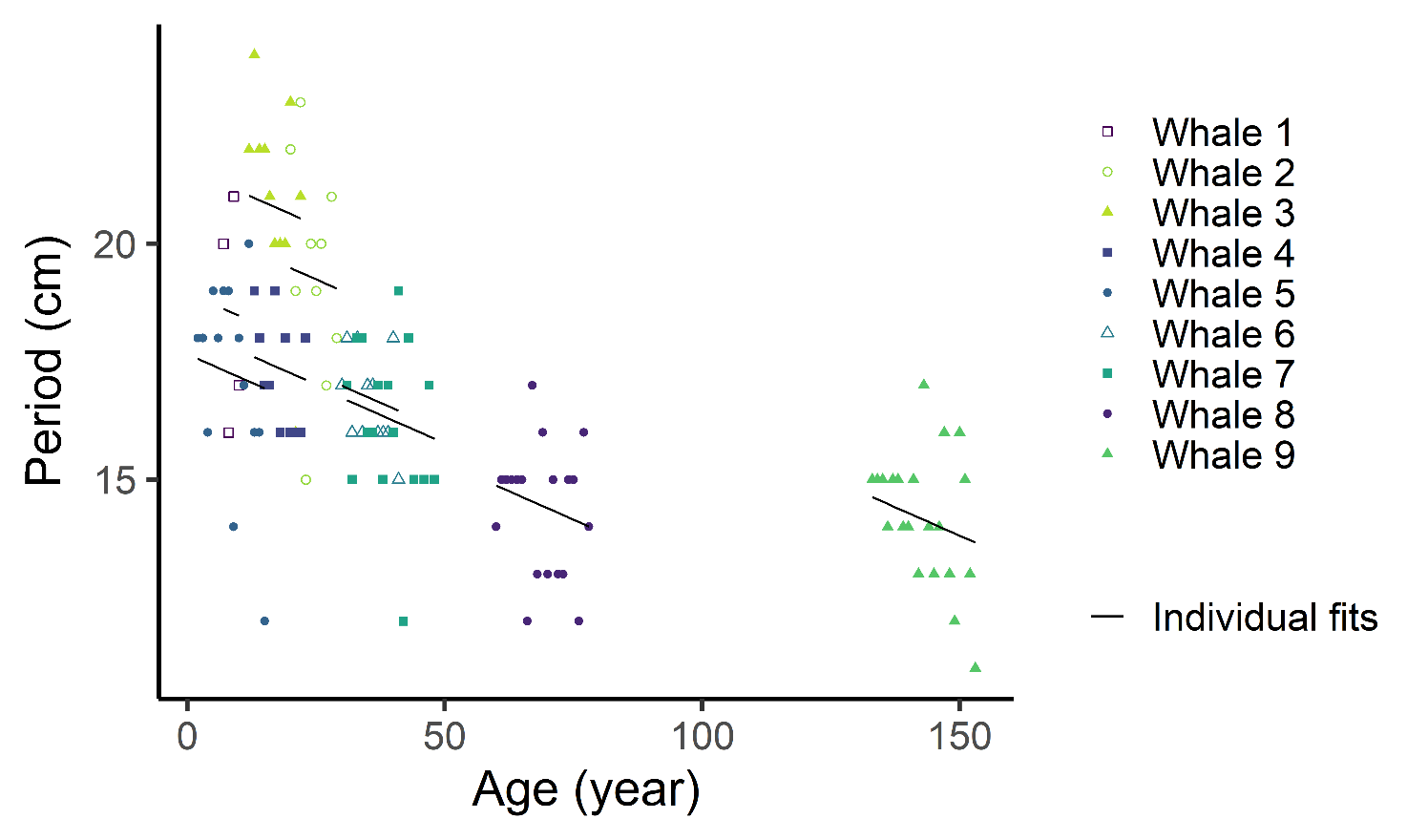


**Figure S11:** Individual fits (lines) estimated by a random intercept linear mixed effects model fit to annual testosterone cycle periods across baleen of nine different-aged male bowhead whales. This model was fit to ages estimated using body length-at-age relationships only, in comparison to the same model fit to ages estimated using a combination of aspartic acid racemization (AAR) and length-at-age relationships (see Figure S9; Table S3)

| **Table S1.** Candidate linear mixed effects models of testosterone cycle periods across baleen of Eastern Canada-West Greenland male bowhead whales (*B. mysticetus*) and estimated whale age. The random intercept model (bold), which was selected as the optimal model based on AICc values, indicate T period (cm) declines with age (year). | | | | | |
| --- | --- | --- | --- | --- | --- |
| **Model** | **logLik** | **AICc** |  |  |  |
| *Null*  T period ~ 1, random = ~1\|whale | –245.0 | 496.1 |  |  |  |
| *Random Intercept and Slope*  T period ~ age, random = ~1+age\|whale | –236.5 | 485.7 |  |  |  |
| ***Random Intercept***  **T period ~ age, random = ~1\|whale** | **–236.9** | **482.2** |  |  |  |
|  | **Estimate** | **SE** | **t-value** | **df** | **p-value** |
| **intercept** | **19.2** | **0.67** | **28.8** | **109** | **<0.001** |
| **age** | **–0.0445** | **0.010** | **–4.43** | **109** | **<0.001** |
|  | **intercept** |  |  |  |  |
| Whale 1 | **19.1** |  |  |  |  |
| Whale 2 | **19.9** |  |  |  |  |
| Whale 3 | **21.4** |  |  |  |  |
| Whale 4 | **18.2** |  |  |  |  |
| Whale 5 | **18.0** |  |  |  |  |
| Whale 6 | **18.5** |  |  |  |  |
| Whale 7 | **18.1** |  |  |  |  |
| Whale 8 | **19.1** |  |  |  |  |
| Whale 9 | **20.5** |  |  |  |  |

| **Table S2.** Linear mixed effects models (random intercept and slope) of peak testosterone concentrations measured across baleen of Eastern Canada-West Greenland male bowhead whales (*B. mysticetus*) and estimated whale age. The table compares parameter estimates of the same model fit to ages estimated using two approaches: a combination of estimates based on aspartic acid racemization (AAR) and body length-at-age relationship (top), and only length-at-age relationships (bottom). AICc values indicated the random intercept and slope model was the optimal model in both cases (over null and random intercept models), and overall parameter estimates for the fixed effect (age) indicate similar declines in peak T (ng g^-1^) with age (year) whether AAR + length-based or just age-based estimates were used (shaded blue). For the most part, individual rates of decline were similar whether AAR + length-based or just age-based estimates were used; however, differences were noted for whales 2 (switch from negative to positive slope) and 4 (slope increased by an order of magnitude) (shaded pink). | | | | | | | |  |
| --- | --- | --- | --- | --- | --- | --- | --- | --- |
| **Age estimation** | **Model** |  |  |  |  |  |  |  |
| ***AAR +*** | ***Random Intercept and Slope*** |  |  |  |  |  |  |  |
| ***Length*** | **Peak T ~ age, random = ~1+age\|whale** |  | **Estimate** | **SE** | **t-value** | **df** | **p-value** |  |
|  |  | **intercept** | **23.1** | **11.2** | **2.06** | **116** | **0.042** |  |
|  |  | **age** | **–0.163** | **0.082** | **–1.98** | **116** | **0.050** |  |
|  |  | **intercept** | **age** |  |  |  |  |  |
|  | Whale 1 | **3.32** | **–0.0215** |  |  |  |  |  |
|  | Whale 2 | **2.93** | **–0.0118** |  |  |  |  |  |
|  | Whale 3 | **13.8** | **–0.128** |  |  |  |  |  |
|  | Whale 4 | **6.96** | **0.00804** |  |  |  |  |  |
|  | Whale 5 | **4.73** | **–0.0434** |  |  |  |  |  |
|  | Whale 6 | **14.1** | **–0.158** |  |  |  |  |  |
|  | Whale 7 | **5.69** | **–0.0351** |  |  |  |  |  |
|  | Whale 8 | **56.6** | **–0.450** |  |  |  |  |  |
|  | Whale 9 | **99.97** | **–0.625** |  |  |  |  |  |
| ***Length*** | ***Random Intercept and Slope*** |  |  |  |  |  |  |  |
|  | **Peak T ~ age, random = ~1+age\|whale** |  | **Estimate** | **SE** | **t-value** | **df** | **p-value** |  |
|  |  | **intercept** | **23.1** | **10.6** | **1.99** | **116** | **0.049** |  |
|  |  | **age** | **–0.159** | **0.089** | **–1.79** | **116** | **0.078** |  |
|  |  | **intercept** | **age** |  |  |  |  |  |
|  | Whale 1 | **3.10** | **–0.00466** |  |  |  |  |  |
|  | Whale 2 | **2.62** | **0.00677** |  |  |  |  |  |
|  | Whale 3 | **14.4** | **–0.159** |  |  |  |  |  |
|  | Whale 4 | **5.67** | **0.0802** |  |  |  |  |  |
|  | Whale 5 | **4.35** | **–0.0448** |  |  |  |  |  |
|  | Whale 6 | **15.2** | **–0.204** |  |  |  |  |  |
|  | Whale 7 | **5.53** | **–0.0309** |  |  |  |  |  |
|  | Whale 8 | **39.9** | **–0.450** |  |  |  |  |  |
|  | Whale 9 | **99.4** | **–0.621** |  |  |  |  |  |

| **Table S3.** Linear mixed effects models (random intercept) of testosterone cycle periods across baleen of Eastern Canada-West Greenland male bowhead whales (*B. mysticetus*) and estimated whale age. The table compares parameter estimates of the same model fit to ages estimated using two approaches: a combination of estimates based on aspartic acid racemization (AAR) and body length-at-age relationship (top), and only length-at-age relationships (bottom). AICc values indicated the random intercept model was the optimal model in both cases (over null and random intercept and slope models), and overall parameter estimates for the fixed effect (age) indicate similar decreases in period (cm) with age (year) whether AAR + length-based or just age-based estimates were used (shaded blue).. | | | | | | | |  |
| --- | --- | --- | --- | --- | --- | --- | --- | --- |
| **Age estimation** | **Model** |  |  |  |  |  |  |  |
| ***AAR +*** | ***Random Intercept and Slope*** |  |  |  |  |  |  |  |
| ***Length*** | **T Period ~ age, random = ~1\|whale** |  | **Estimate** | **SE** | **t-value** | **df** | **p-value** |  |
|  |  | **intercept** | **19.2** | **0.67** | **28.8** | **109** | **<0.001** |  |
|  |  | **age** | **–0.0445** | **0.010** | **–4.43** | **109** | **<0.001** |  |
|  |  | **intercept** |  |  |  |  |  |  |
|  | Whale 1 | **19.1** |  |  |  |  |  |  |
|  | Whale 2 | **19.9** |  |  |  |  |  |  |
|  | Whale 3 | **21.4** |  |  |  |  |  |  |
|  | Whale 4 | **18.2** |  |  |  |  |  |  |
|  | Whale 5 | **18.0** |  |  |  |  |  |  |
|  | Whale 6 | **18.5** |  |  |  |  |  |  |
|  | Whale 7 | **18.1** |  |  |  |  |  |  |
|  | Whale 8 | **19.1** |  |  |  |  |  |  |
|  | Whale 9 | **20.5** |  |  |  |  |  |  |
| ***Length*** | ***Random Intercept and Slope*** |  |  |  |  |  |  |  |
|  | **T Period ~ age, random = ~1\|whale** |  | **Estimate** | **SE** | **t-value** | **df** | **p-value** |  |
|  |  | **intercept** | **19.1** | **0.783** | **24.4** | **109** | **<0.001** |  |
|  |  | **age** | **–0.0481** | **0.089** | **–3.73** | **109** | **<0.001** |  |
|  |  | **intercept** |  |  |  |  |  |  |
|  | Whale 1 | **19.0** |  |  |  |  |  |  |
|  | Whale 2 | **20.4** |  |  |  |  |  |  |
|  | Whale 3 | **21.6** |  |  |  |  |  |  |
|  | Whale 4 | **18.2** |  |  |  |  |  |  |
|  | Whale 5 | **17.7** |  |  |  |  |  |  |
|  | Whale 6 | **18.4** |  |  |  |  |  |  |
|  | Whale 7 | **18.2** |  |  |  |  |  |  |
|  | Whale 8 | **17.8** |  |  |  |  |  |  |
|  | Whale 9 | **21.0** |  |  |  |  |  |  |
